# Development and pilot testing of a risk assessment checklist for children of incarcerated parents in Hong Kong

**DOI:** 10.3389/fsoc.2026.1936866

**Published:** 2026-09-24

**Authors:** Andrey Chau, Wan Yin Yiu, Chloe Iseul Laurence Bailly, Yat Lui Fung, Sandra Kit Man Tsang, Celia Hoi Yan Chan, Paul Wai Ching Wong

**Affiliations:** 1 Department of Social Work and Social Administration, Faculty of Social Sciences, University of Hong Kong, Pokfulam, Hong Kong SAR, China; 2 Department of Social Work, Melbourne School of Health Sciences, Faculty of Medicine, Dentistry and Health Sciences, The University of Melbourne, Melbourne, VIC, Australia

**Keywords:** biopsychosocial model, children of incarcerated parents, ecological systems theory, Hong Kong, parental incarceration, risk assessment, social work

## Abstract

Children of incarcerated parents (CIP) are exposed to a range of biological, psychological, and social risks, yet existing risk assessment tools do not adequately capture incarceration-specific experiences. This article details the development of a comprehensive, theory-driven risk assessment checklist tailored to CIP aged 6–11, with consideration of the sociocultural context of Hong Kong. The checklist was developed using an integrative framework combining ecological systems theory and the biopsychosocial model. Items were adapted from validated instruments and supplemented with incarceration-specific risk factors identified in prior literature. The tool was iteratively refined through professional review, expert consultation, and pilot testing with frontline social workers. Key design features included a trauma-informed approach, bilingual versions (Chinese and English), age-appropriate formats for children and their caregivers, and a traffic-light scoring system to guide risk classification and follow-up actions. The resulting tool captures multidimensional family-level and context-specific risks, including disrupted caregiving arrangements, stigma, and institutional barriers to parent–child contact. Pilot implementation provided preliminary feedback on the tool’s feasibility and acceptability in real-world social service settings. The tool is intended to support systematic risk identification and inform timely intervention. However, its effectiveness and validity require further evaluation. These findings suggest that the checklist addresses a critical gap in existing assessment practices by providing a structured and contextually relevant tool for CIP. The article highlights the value of integrating theoretical frameworks with local contextual factors in instrument development. Further research is needed to establish the checklist’s psychometric properties and evaluate its applicability across diverse family and service settings.

## 1 Introduction

Parental incarceration is a significant family stressor with consequences that extend beyond the individual child to caregiving arrangements, household stability, and intergenerational relations. It is associated with adverse outcomes for children of incarcerated parents (CIP), including externalising (e.g., aggression, substance use) and internalising behaviour problems (e.g., anxiety, depression) across developmental stages (Antle et al., 2020; Austin et al., 2022; Luk et al., 2023). Despite being recognised as a high-risk population, risk assessment tools tailored to the circumstances of these children remain limited. This gap reflects the diversity of their backgrounds and experiences, which complicates the development of a standardised tool usable across contexts. For instance, family fragmentation resulting from incarceration makes longitudinal research difficult.

Timely identification of risk is essential because parental incarceration is associated with a higher risk for children’s broader well-being outcomes and the incarceration period may offer a practical opportunity for intervention. A longitudinal family study posited that the “point of incarceration” is an opportunity for early intervention and the provision of age-appropriate social services to address unmet material needs, residential instability, and behaviour problems (Geller et al., 2009). A recent scoping review similarly highlighted the need for developmentally targeted services and systems that better capture parental status such that CIP and families receive needed support (Obus et al., 2024). Therefore, risk assessment is a clinical task and a mechanism by which families gain access to care, resources, and institutional support.

Existing risk assessment instruments, such as the Child Behavioural Checklist (Achenbach, 1991), Child Stress Disorder Checklist (Bosquet Enlow et al., 2010) or Childhood Trauma Questionnaire–Short Form (Bernstein et al., 2003), assess general aspects of child well-being but do not capture the unique risks associated with parental incarceration. These risks include exposure to parental arrest or sentencing, as well as the consequences of such experiences (Dallaire et al., 2015). For example, exposure to parental arrest has been associated with blunted stress responses and stronger internalising and externalising behaviour problems (Muentner et al., 2021; Metcalfe et al., 2023). More broadly, children in this population often exhibit emotional and behavioural issues, educational challenges, socioeconomic disadvantages, and unstable home environments (Geller et al., 2009; Poehlmann-Tynan et al., 2018). Despite this evidence, there is currently a lack of incarceration-specific risk assessment tools that integrate these domains for use in frontline practice, especially in an Asian context. These findings underscore the need for a targeted, comprehensive risk assessment tool to facilitate earlier risk identification and the provision of responsive support for CIP.

Given the need for this assessment tool, an important consideration is the age range for which direct screening would be most feasible and informative. Middle childhood was selected as the focus for practical and developmental reasons. Child-health measurement research supports the feasibility of child self-report from approximately age 6 onward, provided that questionnaires use developmentally appropriate language, response options, and administration procedures. Support is strongest for school-age children, as younger children (age 5 or under) may require simplified or visually supported formats and additional assessment of response quality (Bevans et al., 2020; Coombes et al., 2021). Accordingly, while direct screening of children’s experiences is feasible in this age range, responses should be interpreted alongside caregiver or professional observations. Developmentally, the 6 to 11 year period coincides with primary school entry and the consolidation of basic academic skills, peer relationships, and emotion regulation. These domains are relevant to CIP as suggested by a population-based study, where parental offending was associated with difficulties that either emerged, remitted, or persisted between ages 5 and 11, with the strongest association observed for persisting conduct problems (Whitten et al., 2019). Furthermore, a developmental systematic review suggested that the associations between parental incarceration and child well-being appear more pronounced for children aged 7 to 11, supporting this period as a pragmatic target for initial screening (Herreros-Fraile et al., 2023). Early identification during this period thus offers an opportunity to intervene before problems become more pronounced in adolescence, when risk-taking and peer influences intensify. Together, these findings suggest that middle childhood represents a developmentally sensitive and practically feasible window for screening and early intervention among CIP. As a first step, the present checklist therefore concentrates on middle childhood, while recognising that separate tools or thresholds may be needed for younger children and adolescents.

### 1.1 Biological and health-related impact of CIP

Across their developmental trajectory, CIPs are at increased risk of biological and physiological dysregulation relative to their peers without a history of parental incarceration. These include higher allostatic load, accelerated cellular ageing, and systemic inflammation (Mitchell et al., 2017; Niño and Cai, 2020; Austin et al., 2022). They are also more likely to present with physical health problems such as elevated cholesterol, asthma, and migraines (Lee et al., 2013).

These risks may vary across developmental stages. For children aged 0 to 6, domestic violence by the father before incarceration was found to moderate the relationship between paternal incarceration and child physical health outcomes (Wildeman, 2012). Among children aged 7 to 11, parental incarceration has been linked to poorer sleep and dietary patterns, including short sleep duration and greater consumption of salty snacks, starches, sweets, soda, and fast food (Jackson and Vaughn, 2017). Although observed differences may reflect variation in study samples, designs, or measures rather than differences between developmental stages, they may nonetheless provide provisional guidance when considering age-related risks. Notably, differences have also been identified between paternal and maternal incarceration: paternal incarceration is more strongly associated with sleep problems and starch consumption, while maternal incarceration is associated with greater fast-food consumption (Jackson and Vaughn, 2017).

### 1.2 Psychological and behavioural impact of CIP

Parental incarceration has significant psychological impacts on children and adolescents, often in relation to attachment disruption, separation distress, and developmental delays, especially when contact with the incarcerated father is limited (Dallaire et al., 2012; Arditti and Savla, 2013; Warren et al., 2019). Aside from internalising behaviour problems, studies have found associations between parental incarceration and externalising symptoms, including aggression and attention difficulties (Geller et al., 2011; Austin et al., 2022; Luk et al., 2023).

Gender differences have also been observed, with boys more likely to display externalising behaviours and girls more likely to exhibit internalising behaviours (Warren et al., 2019). These associations appear particularly pronounced among children aged 7 to 11, a developmental period characterised by increasing social and academic demands (Herreros-Fraile et al., 2023).

Beyond psychological impacts, several behavioural risks have been identified, including increased delinquency, family victimisation, and antisocial behaviour. These risks are further exacerbated in the presence of adverse family dynamics, such as poor caregiver-child relationships, sibling conflict, interparental discord, or negative parental communication (Aaron and Dallaire, 2009; Davis and Shlafer, 2017). There is also evidence of intergenerational continuity, as maternal substance abuse has been shown to predict incarceration in adult children (Dallaire, 2007).

### 1.3 Social and educational impact of CIP

Parental incarceration also disrupts children’s social environments through parent–child separation, unstable caregiving, and financial hardship (Thorne et al., 2023; Obus et al., 2024). These factors may undermine attachment security and disrupt daily routines, affecting both academic functioning and peer relationships (Murray et al., 2012).

Regarding educational outcomes, paternal incarceration has been associated with poorer academic performance, including poorer math problem-solving skills, greater attention and memory difficulties, as well as increased likelihood of grade repetition and school suspension (Herreros-Fraile et al., 2023). Additionally, social stigma in school settings may contribute to feelings of isolation and discourage children from disclosing their parental incarceration status (Warren et al., 2019).

Gender-specific patterns have also been observed. For boys, parental incarceration has been linked to poorer non-cognitive school readiness and a greater likelihood of special education placement (Haskins et al., 2014). For girls, paternal incarceration has been associated with lower reading comprehension and math problem-solving scores (Herreros-Fraile et al., 2023).

### 1.4 Hong Kong culture and context

Findings from Hong Kong largely align with those reported in Western contexts, including associations between parental incarceration and academic stress, psychological distress, and stigma-related concealment among children aged 9 to 11 (Yau and Chung, 2014). Caregiver mental health has also been identified as a key mediator linking perceived stress to children’s behavioural problems (Chui, 2016). Yet, several cultural considerations highlight the importance of contextualising risk assessment for CIP in Hong Kong.

Traditional Chinese values emphasise family harmony, clearly defined social roles and obligations, and the prioritisation of collective interests over individual ones (Shek et al., 2022). Within this framework, parental incarceration represents a profound disruption to family structure and undermines traditional parental authority, while also extending courtesy stigma to the entire family unit (Wells, 2020). Parenting practices in Hong Kong, often characterised by strong parental control and a focus on academic achievement (Kobakhidze et al., 2024), may further shape how children experience and respond to parental incarceration. In addition, a previous study involving Hong Kong parents revealed persistent traditional and gender-stereotyped expectations for sons and daughters, such as a preference for sons and expectations for daughters to be homemakers. These expectations may influence how children experience family disruption and highlight the importance of considering potential gender differences in their responses to parental incarceration (Shek et al., 2019).

Caregiving arrangements also warrant consideration. In many families, caregiving responsibilities are taken up by grandparents or domestic helpers, reflecting parents’ work demands and the high cost of living. While these arrangements may provide stability, they may introduce inconsistencies in caregiving practices, disrupt attachment relationships, and reflect intergenerational differences in child-rearing expectations (Poehlmann, 2003; Shek et al., 2022). In families affected by parental incarceration, the quality and continuity of substitute caregiving are especially important because children’s adjustment is shaped not only by parental absence but also by the everyday social relations through which care is organised (Cecil et al., 2008). Prior research has also found that caregiver parenting is associated with improvements in children’s internalising and externalising symptoms across 6 months (Berkel et al., 2023). Stable and nurturing caregiving can support more secure attachment relationships, whereas fragmented or inconsistent care may heighten emotional insecurity and adjustment difficulties (Poehlmann, 2005).

Education in Hong Kong is highly competitive and often associated with substantial academic pressure. In many families, there is a strong emphasis on academic achievement, which may lead parents and caregivers to prioritise school performance over emotional well-being, peer relationships, and play (Cho and Chan, 2020). For CIP, this pressure may intensify existing stressors, interact with other challenges, and make it more difficult to cope with family disruption (Thorne et al., 2023). As a result, parental incarceration not only affects family life, but also educational experiences and sense of belonging within school settings.

Together, these cultural and contextual factors highlight the importance of developing assessment tools that are sensitive to the local service environment.

### 1.5 Purpose of the present work

While existing literature demonstrates that CIP face risks across biological, psychological, and social domains, to our knowledge, there is a lack of assessment tools that measure incarceration-specific factors for this population. To address the research and practice gaps, we developed a theory-driven and comprehensive risk assessment checklist for children aged 6 to 11 with incarcerated parents. This article describes the development of the Checklist.

Development was guided using two complementary frameworks: ecological systems theory (Bronfenbrenner, 1977) and the biopsychosocial model (Engel, 1977). Together, these frameworks provided a structured basis for integrating evidence on risk and protective factors associated with parental incarceration. This tool is intended to help social workers identify risk factors more systematically, support timely and targeted intervention, and improve the consistency of professional judgement. This work was developed in partnership with a non-governmental organisation (NGO) serving justice-involved populations, including CIP and their families, to ensure relevance and practicality for frontline use.

## 2 Materials and methods

### 2.1 Checklist design and literature review

Development of the risk assessment checklist (referred to as “Checklist” hereafter) began with a literature review on parental incarceration to identify relevant risk factors and existing assessment tools.

The Checklist was informed by a targeted, iterative review of the literature on the impacts of parental incarceration on children. Searches were conducted through databases such as PsycINFO, Scopus, and Web of Science, and through Google Scholar using combinations of terms such as parental incarceration, parental imprisonment, incarcerated parents, children of incarcerated parents, children of prisoners, child outcomes, and neglected children. Initially, the scope of studies focused on children under age 18 with at least one incarcerated parent, without restricting inclusion according to the duration of parental incarceration. Given the limited evidence base, particularly in Asia, potentially relevant studies were considered broadly. For example, studies involving adult offspring were also consulted where they provided insight into the long-term consequences of parental incarceration.

Two relevant systematic reviews, one focused on Asia and one covering broader international evidence, provided an initial basis for identifying the principal risk domains associated with parental incarceration, including biological and health-related, psychological and behavioural, social and familial, and educational risks (Herreros-Fraile et al., 2023; Luk et al., 2023). At the same time, two conceptual frameworks (ecological systems theory and the biopsychosocial model) were selected to organise these domains and structure the Checklist content. Additional targeted searches, primarily through Google Scholar, were then conducted to identify risk assessment instruments used with CIP or in a Chinese context. As there was a lack of instruments designed for CIP, the search was expanded to include related assessment tools for children. The identified instruments were compared in terms of their constructs, target populations, administration procedures, and scoring approaches.

Findings from the literature review were discussed iteratively by the research team and frontline social workers, incorporating both research and practice perspectives. Items were compiled from several established tools and adapted to better capture the circumstances of CIP, with particular attention to family organisation, caregiving arrangements, and context-specific risks. These items were reviewed and refined iteratively to produce the initial set. This process was intended to support theory-informed and practice-relevant item development; it was not conducted as a formal systematic or scoping review and did not involve a registered protocol or formal risk-of-bias assessment.

The Checklist comprised eight sections covering caregiver information, child characteristics, parental background, family mental health and incarceration history, home observations, and risk assessments for both caregivers and children (Table 1).

**Table 1:** Overview of the checklist sections.

Section	Parent/primary caregiver version	Child version
1	Caregiver basic information	
2	Child’s personal information	
3	Parental background information	
4	Mental health history of family members	
5	Incarceration history of family members	
6	Social worker home visit observations	
7	Risk assessment checklist (parent/primary caregiver version)	
8		Risk assessment checklist (child version)

### 2.2 Theoretical and contextual foundations

Two complementary theoretical frameworks guided Checklist development to ensure a comprehensive and conceptually grounded approach: (i) ecological systems theory (Bronfenbrenner, 1977) and (ii) the biopsychosocial model (Engel, 1977). In addition, the sociocultural context of Hong Kong was incorporated to enhance the ecological validity and practical relevance in service settings.

#### 2.2.1 Ecological systems theory

Bronfenbrenner (1977) ecological systems theory conceptualises child development as occurring within nested systems that dynamically shape individual experiences and developmental outcomes. This theory has been previously applied to understand the impact of parental incarceration on children (Holmes et al., 2010), illustrating its usefulness for understanding the effects of parental incarceration on family life and care relations.

At the microsystem level, parental incarceration often disrupts attachment and alters family routines, affecting children’s emotional security. The quality of interactions with remaining caregivers, siblings, and peers becomes critical in either mitigating or exacerbating these disruptions. At the mesosystem level, effective communication and consistency across settings such as home, school, and community are vital for child well-being. For CIP, coordination among social services, schools, and family can either buffer or exacerbate risks, depending on the level of collaboration. At the exosystem level, children are indirectly influenced by institutional environments such as correctional facilities and social service systems. Decisions within these settings, including visitation policies and service accessibility, can significantly affect children’s opportunities for maintaining relationships and receiving support. At the macrosystem level, broader societal factors, including cultural norms, public attitudes towards incarceration, and policy frameworks, shape how CIP are perceived and treated, often contributing to stigma, social exclusion, and reduced access to resources.

Integrating ecological systems theory into the Checklist’s design enables a holistic assessment of children’s environments, ensuring that risk identification extends beyond individual-level factors to encompass family dynamics, institutional structures, and sociocultural influences.

#### 2.2.2 Biopsychosocial model

The biopsychosocial model (Engel, 1977) complements the ecological perspective by providing a multidimensional framework for assessing individual functioning across biological, psychological, and social domains. This approach is well suited to CIP, whose experiences often reflect combined effects of disrupted care, family stress, and social disadvantage.

At the biological level, the model encompasses physical health and developmental factors, including malnutrition, sleep disruption, and stress-related health conditions. It also considers risks arising from unstable caregiving environments, such as neglect, poor hygiene, and limited access to healthcare services. At the psychological level, the model focuses on emotional and cognitive functioning, including symptoms of depression and anxiety, emotional dysregulation, and coping difficulties associated with parental separation and stigma. At the social level, it examines children’s interactions within their immediate and broader social networks, where experiences of stigma, bullying, and social isolation may hinder social development and compound psychological distress.

Together, these domains provide a structured framework for identifying and categorising the multidimensional risks faced by CIP, thereby informing more targeted and integrated interventions.

#### 2.2.3 Local context in Hong Kong

The development of the Checklist also incorporated sociocultural characteristics specific to Hong Kong, recognising that contextual factors may shape both risk and intervention strategies.

First, institutional practices related to incarceration may constrain parent–child contact. For example, physical barriers such as glass partitions during prison visits can limit opportunities for meaningful interaction and emotional bonding. Second, caregiving arrangements in Hong Kong often differ from those in Western contexts. CIP are frequently cared for by a combination of grandparents and domestic helpers, reflecting broader socioeconomic conditions and labour patterns. While these arrangements may provide continuity of care, they may also introduce variability in parenting practices and attachment experiences. Third, Hong Kong’s education system is typically rigorous and stressful. For CIP, this may compound existing stressors and affect their school experiences.

By integrating these localised factors, the Checklist is designed to capture the lived experiences of CIP in Hong Kong more accurately and to support contextually appropriate assessment and intervention strategies.

### 2.3 Design considerations

#### 2.3.1 Trauma-informed approach

A trauma-informed approach was adopted to ensure that the Checklist is sensitive to the experiences of CIP, particularly when addressing potentially distressing topics such as abuse, neglect, or sexual assault. Item wording was carefully refined by experts (including clinical psychologists, social workers, researchers, a paediatrician) and after pilot trials to minimise the risk of re-traumatisation while maintaining conceptual clarity. In addition, social workers at our NGO partner administering the Checklist were supported by specialised training to recognise and manage participants’ emotional responses during assessment.

#### 2.3.2 Bilingual accessibility

To ensure accessibility in Hong Kong, the Checklist was developed in both Chinese and English (see Supplementary materials S1, S2 files respectively). The translation process involved close collaboration with clinical psychologists, a paediatrician, and several experienced social workers to preserve conceptual equivalence and cultural nuance across languages. Particular attention was given to avoiding ambiguous or culturally inappropriate phrasing, thereby enhancing clarity and reliability in administration.

#### 2.3.3 Age-appropriate versions

For social workers to assess and compare responses between parties, Sections 7 and 8, which comprise the core risk assessment items, include parallel versions for parents or primary caregivers and for children aged 6 to 11. Language was tailored and simplified to ensure comprehension and age-appropriateness for CIP. As cognitive and linguistic abilities vary across this age range, social workers were instructed to provide clarification or rephrasing when needed to ensure accurate understanding and responses.

### 2.4 Initial item selection

Initial item selection was guided by the literature on family risk and by established instruments relevant to child well-being. Although several existing tools are used with children aged 6 to 11, none sufficiently capture the specific challenges associated with parental incarceration, including changes in care arrangements and socioeconomic instability. Items were adapted from validated measures, including the Child Behavioural Checklist (Achenbach, 1991), Child Stress Disorder Checklist (Bosquet Enlow et al., 2010), Strengths and Difficulties Questionnaire (Kersten et al., 2016), Childhood Trauma Questionnaire–Short Form (Bernstein et al., 2003), and Reactive-Proactive Aggression Questionnaire (Raine et al., 2006).

Item selection and adaptation were conducted through internal review meetings to ensure conceptual relevance and contextual appropriateness. This process produced a comprehensive item pool capturing multiple domains, including home environment, behavioural and emotional functioning, and incarceration-specific situational risks.

### 2.5 Professional review and iterative refinement

The Checklist underwent iterative refinement through multiple stages of professional review, pilot testing, and expert consultation. Feedback from frontline practitioners and clinical experts was used to enhance item clarity, cultural relevance, and practical usability. Revisions included removing redundant or ambiguous items, refining translations, and adjusting item distribution across respondent versions.

Table 2 summarises the refinement process, including changes in item number across parent/caregiver and child versions. The final versions of the Checklist in Chinese and English are provided in Supplementary material S1, S2 files, respectively.

**Table 2:** Summary of checklist refinement process.

Stage	Input source	Key changes	No. of items (parent/caregiver version)	No. of items (child version)
1	Initial professional review (NGO social workers)	Expanded item pool to improve relevance and coverage for CIP; addressed gaps in social and population relevance	87	105
2	Internal review and refinement	Removed redundant and hard-to-translate items; streamlined categories to key items	68	65
3	Clinical psychologist review and translation	Refined Chinese translation, resolved inconsistencies, adjusted item distribution (some retained only in child version)	56	64
4	Pilot testing and expert feedback	Further revisions to improve clarity, applicability, and alignment with current practice needs	58	64
5	Peer review process	Further revisions to improve item selection and scoring thresholds	59	65

### 2.6 Scoring instructions

Sections 2, 6, 7, and 8 of the Checklist address core risks and employ a traffic-light scoring system (Red, Yellow, Green) similar to that used in recently developed clinical risk assessments (Vanburen et al., 2022; Wawra-Hehenberger et al., 2025). Responses are recorded in a dichotomous (Yes/No) format and subsequently mapped onto these three levels to guide interpretation, intervention planning, or other risk-management activities (Table 3).

**Table 3:** Summary of scoring instructions.

Risk level	Risk level meaning	Follow-up actions
Red	High: Checklist responses indicate service needs requiring immediate attention.	Conduct independent assessment and case follow-up as needed.
Yellow	Moderate: Checklist responses suggest potential risks requiring further clarification.	Gather additional information to assess risk level and provide appropriate support.
Green	Low: No potential risks or immediate needs identified.	No follow-up required at this time.

Initial coding decisions (i.e., the assignment of items to Red, Yellow, or Green categories) were informed by the clinical and research expertise of the internal research team. The proposed scoring thresholds were subsequently reviewed by external experts as part of the Checklist development process. This structured scoring approach is intended to support consistent decision-making, facilitate timely and proportionate responses to identified risks, and strengthen professional judgement. Detailed Chinese and English scoring instructions are provided in Supplementary materials S3, S4.

## 3 Results

### 3.1 Pilot testing

Pilot testing took place between May 2025 and November 2025 to establish the Checklist’s content validity. During this period, six NGO social workers, a few of whom were involved in the Checklist development process, implemented the Checklist with families impacted by current or previous parental incarceration. A total of 37 families agreed to participate in the trial testing of the Checklist. Table 4 presents an overview of their demographics.

**Table 4:** Demographics of assessed families.

Category	*n*
Assessed parents/caregivers	
Father	3
Mother	20
Grandmother	14
Assessed children	
Male	21
Female	16
Mean age	8.7 (range: 6–11)

### 3.2 Social worker feedback

Feedback from social workers was gathered in a discussion format following the trial period. Overall, the Checklist’s broad coverage was seen as helpful for surfacing issues that are not usually discussed and for supporting future counselling sessions, but several outstanding items warranted further refinement before broader use.

The main concerns were wording, timing, and applicability. Respondents struggled with ambiguous terminology or wording, unclear reference periods, and questions that did not fit every family situation. Children of differing ages often needed language tailored to their developmental levels. For example, younger children had difficulty understanding difficult phrases (e.g., “good for nothing” or “hopeless”), references to suicidal thoughts (e.g., not wanting to wake up), and sexualised humour. In such cases, their lack of understanding cannot be assumed to indicate that the experience had not occurred. The items therefore required clarification by social workers before a response was recorded. If the child continued to indicate that the experience had not occurred, a “No” response would be recorded.

Implementation challenges were also identified. Social workers noted that parental presence during Checklist completion may influence children’s responses, raising concerns about reliability. Even with separated parent/primary caregiver and children’s versions, social workers may complete the assessment during home observations, where separation of parents and children may not always be possible. Several sections would benefit from clearer instructions or more flexible phrasing and guidance on how to address sensitive topics. In addition, established trust between social workers and families may encourage more honest responses and improve the accuracy of the Checklist. To support more reliable completion, it was suggested that the Checklist be administered gradually over a month rather than in a single session, allowing rapport to develop without excessive delay.

### 3.3 Expert feedback

The internal research team included two clinical psychologists who were involved throughout the development process and iteratively provided feedback. Their expertise in special educational needs, as well as children and youth mental health, informed feedback on wording, developmental appropriateness, conceptual clarity, scoring thresholds, and the practicality of the Checklist for frontline use.

Two external professionals with expertise in child safeguarding (i.e., a senior social worker and a paediatrician), who were not involved in the development process, were invited to provide expert feedback on two separate occasions (November 2025 and February 2026). Their feedback focused primarily on wording refinements, including ensuring that the revised Checklist aligned with the newly established Mandatory Reporting of Child Abuse regulations in Hong Kong during January 2026, improving the applicability of questions across different family situations and contexts, and suggestions for amendments to scoring thresholds.

## 4 Discussion

The present work developed a theory-driven and contextually grounded risk assessment checklist for CIP aged 6 to 11. The Checklist addresses a broader sociological concern: how family life, care arrangements, and children’s well-being are reshaped when parental incarceration disrupts everyday routines and relationships. Several key contributions emerge from the development process and the final structure of the tool.

First, the Checklist addresses a critical gap in existing assessment tools by incorporating incarceration-specific risk factors not captured by general child well-being measures. While widely used instruments capture general aspects of child functioning, they do not adequately reflect experiences unique to CIP, such as parental arrest exposure, disrupted caregiving arrangements, stigma-related stress, and changes in family organisation. By integrating these dimensions, the Checklist offers a more context-sensitive understanding of how incarceration affects family life and children’s access to care.

Second, the integration of ecological systems theory and the biopsychosocial model supports a multi-level understanding of risk. This dual-framework approach enables practitioners to assess risk across interacting domains, including individual functioning, family dynamics, institutional influences, and broader sociocultural contexts. As a result, the Checklist moves beyond a narrow symptom focus and facilitates a more holistic account of children’s lived experiences and the structures shaping them.

Third, incorporating Hong Kong-specific sociocultural factors enhances the Checklist’s ecological validity and sociological relevance. Elements such as constrained visitation practices, reliance on domestic helpers and extended family caregiving, and culturally embedded stigma highlight the local organisation of care and social meanings attached to incarceration. This localisation strengthens both the theoretical basis and the practical relevance of the tool within the service context.

Finally, the structured scoring system and trauma-informed design were developed to support the Checklist’s potential utility in frontline practice. The traffic-light classification provides a preliminary framework to support timely and proportionate responses to identified concerns. As the Checklist’s reliability, validity, feasibility, and effects on professional decision-making and service delivery have yet to be established, these features should be viewed as design strengths that may support practical application rather than as evidence of clinical utility at this stage.

### 4.1 Strengths and limitations

A key strength of the present work is its theory-driven and multidisciplinary approach, which integrates established conceptual frameworks with practitioner-informed insights. Using validated measures as a foundation for item development strengthens conceptual coverage, while the iterative refinement process improved clarity, relevance, and usability. The availability of bilingual versions also increases accessibility and applicability in Hong Kong.

Despite these practical strengths, several methodological and conceptual limitations should be acknowledged. First, pilot testing was conducted at a single site and limited to families receiving support from one NGO, which may restrict generalisability to other service contexts or regions. Second, while certain items were drawn from validated instruments, the Checklist itself has not yet undergone independent validation. As a result, its psychometric properties, including reliability and factor structure, have not yet been empirically established. Third, the focus on children aged 6 to 11 years may not adequately capture the developmental needs of younger children or adolescents, thereby excluding some important subgroups with potentially distinct needs. Fourth, the reliance on self-reported information from both caregivers and children may introduce response biases such as social desirability or the underreporting of sensitive issues. Fifth, children were not consulted in the development of this tool. Their perspectives were instead captured indirectly through social workers’ observations and clinical expertise. Finally, although the binary scoring system enhances usability, it may oversimplify complex family circumstances. It is hoped that training provided to frontline social workers will help them build sufficient rapport and sensitivity to identify concerns that cannot be fully captured through a structured tool.

### 4.2 Future research directions

Future validation and refinement studies would benefit from child and youth consultation to ensure content validity, relevance, and acceptability. An effective assessment tool should identify children at risk without overidentifying those who are not, as false positives may contribute to unnecessary service use and stigma, whereas false negatives may delay preventive support. Studies should also test the Checklist in more diverse service settings and geographical contexts. Broader sampling would help establish whether the tool remains useful across different organisational environments and family situations. Formal psychometric evaluation is also needed to determine reliability, validity, and underlying structure.

Further work should examine whether the Checklist can be adapted for younger children and adolescents, whose care needs may differ. It would also be valuable to explore how the tool functions in schools, healthcare settings, and community organisations, where children and families may encounter different forms of support. Qualitative follow-up research could further clarify how caregivers and children interpret incarceration-related risks and how these risks are embedded in everyday family life.

### 4.3 Implications

This Checklist has several implications for research and practice. For researchers, it illustrates how assessment tools can be developed from a sociological understanding of family life, care arrangements, and institutional inequality. For frontline practitioners, it offers a structured way to identify incarceration-related risks and support more timely, targeted intervention for children and their caregivers.

Moreover, this work contributes to the broader field by offering a framework for the development of specialised assessment tools for vulnerable populations. The Checklist may serve as a foundation for future instrument development and adaptation, both within Hong Kong and in other sociocultural contexts, where similar needs exist.

## 5 Conclusion

This article presents the development of a risk assessment checklist for children of incarcerated parents aged 6 to 11, grounded in ecological systems theory and the biopsychosocial model and adapted to the Hong Kong context. By incorporating incarceration-specific risks and family-centred contextual factors, the Checklist provides a structured approach to assessing biological, psychological, and social domains of children’s lives.

The inclusion of contextual considerations, such as visitation constraints and caregiving arrangements, further enhances its relevance to the target population. Overall, the Checklist represents a promising tool for improving risk identification and informing targeted interventions for CIP and their families. Future work should focus on empirical validation and adaptation across developmental stages and cultural contexts to maximise its utility and impact.

## Data Availability

The original contributions presented in the study are included in the article/Supplementary material, further inquiries can be directed to the corresponding author/s.
