# Supplementary material for: Development and pilot testing of a risk assessment checklist for children of incarcerated parents in Hong Kong
Source: Front Sociol. 2026 Sep 24;11:1936866. doi: 10.3389/fsoc.2026.1936866 (PMC13647460; doi:10.3389/fsoc.2026.1936866)
Supplement: Supplementary file 1 [file Supplementary_file_1.DOCX]

Supplementary Material

This file contains the Chinese version of the Risk Assessment Checklist, with a total of 21 pages.

**藍巴士賽馬會結伴成長計劃 - 兒童風險評估檢核表（6–11歲）**

社工姓名：________________ 個案編號：________________ 日期：________________

個案狀況：新個案 / 舊個案（請圈選）

1. **本檢核表之目的及作用**
   1.1. 本檢核表為風險評估工具，旨在更有系統地協助社工辨識兒童（6至11歲）於情緒、心理及生活環境中可能面對的困難。
   1.2. 評估結論的作用
        1.2.1. 總結兒童在情緒、發展、行為、生理和文化方面的風險等級。
        1.2.2. 為社工和照顧者提供具體可行的建議，如轉介諮詢或增加學校支援。
2. **作答方式、版本及分部**
   2.1. 問卷分為家長**/**主要照顧者版本及兒童版本，由社工朗讀問題並根據受訪者的回答填寫。
   2.2. 分為家長**/**主要照顧者版本及兒童兩個版本，分部如下:

|  | **家長/主要照顧者版本** | **兒童版本** |
| --- | --- | --- |
| 1 | 照顧者基本資料 |  |
| 2 | 孩子個人資料 |  |
| 3 | 家長背景資料 |  |
| 4 | 家庭成員精神健康歷史 |  |
| 5 | 家庭成員監禁歷史 |  |
| 6 | 社工家訪觀察 |  |
| 7 | 孩子狀況檢核表（家長/主要照顧者版本） |  |
| 8 |  | 孩子狀況檢核表（兒童版本） |

3. **檢核表之特色**
   3.1. 以理論及中外文獻為基礎。
   3.2. 照顧文化與地域適應性。
        3.2.1. 確保使用符合文化背景的語言。
        3.2.2. 考慮與家庭和監禁相關的社會規範和價值。
        3.2.3. 根據當地情況調整問題。
   3.3. 簡單易明，配合作答者的特性: 年齡及教育程度。
   3.4. 用詞謹慎, 避面刺激作答者的情緒。
4. **社工填寫須知**
   4.1. 建議分開進行家長/主要照顧者與兒童版本的問卷，以保障雙方的私隱與真實性。
   4.2. 家長/主要照顧者須至少已擔任該兒童的照顧職責滿六個月。
   4.3. 要確保訪談地點安靜、舒適、無干擾。
   4.4. 建議於家訪時進行問卷， 以方便了解家居環境。
   4.5. 本問卷預計需時約 1 至 2 小時完成。問卷可於一段時間內分次填寫，但建議盡早完成，例如於個案開展的首一個月內。
   4.6. 兒童版問卷的用詞已調整，符合 6 至 11 歲兒童的理解程度。若兒童對個別詞彙或句子仍有疑惑，社工可視乎情況作即時解釋。
5. **簡介與知情參與**
   5.1. 可用以下語句作簡介：「這份問卷是為了幫助我們更了解您和孩子的情況，讓我們提供更適切的支援。」
   5.2. 強調參與者有權選擇不回答任何問題，亦可要求某些答案不記錄在問卷中。家長/主要照顧者是否參與調查不會影響所獲得的支援服務。
6. **保密與資訊披露原則**
   6.1. 除非根據2026年1月生效的《強制舉報虐待兒童條例》發現有潛在風險，否則社工不會主動向任何人透露答案。
   6.2. 若家長或孩子要求知悉對方的回答內容，社工會依當事人的要求，酌情處理。
   6.3. 若家長要求根據《個人資料(私隱)條例》查閱兒童的資料，則會按相關規定處理。
7. **敏感資訊與高風險情況的處理**
   7.1. 若發現任何涉及危機、傷害或虐待的情況，請即時依循相關法例的規定或機構的保護兒童程序處理。

# 父母／主要照顧者專用問卷

請協助家長或主要照顧者就目標孩子______填寫此問卷，問卷共分為五個部分，包括照顧者基本資料、孩子個人資料、家長背景資料、家庭成員精神健康歷史及家庭成員監禁歷史。請確保答案的真實性，並根據需要解釋問題內容。

# 第一部分：照顧者基本資料

第一部分包括主要照顧者的基本個人背景資料。為確保資訊之代表性，主要照顧者須至少已擔任該兒童的照顧職責滿六個月。

1. 你是孩子的：

□ 父親

□ 母親

□ 祖父/外公

□ 祖母/外婆

□ 其他關係 (請註明)：＿＿＿＿＿＿＿＿＿

2. 性別：

□ 男性

□ 女性

□ 其他

3. 年齡：＿＿＿＿＿＿＿＿＿＿
4. 種族/族裔：

□ 華人

□ 印尼

□ 菲律賓

□ 白人

□ 印度

□ 巴基斯坦

□ 尼泊爾

□ 日本

□ 泰國

□ 其他: 請註明：＿＿＿＿＿＿＿＿＿＿

5. 教育程度：

□ 小學或以下

□ 初中

□ 高中

□ 大專（副學士/文憑/證書）

□ 大學（學士學位）

□ 大學以上

6. 就業狀況：

□ 全職

□ 兼職

□ 自由職業者

□ 待業

□ 退休

□ 持家者（家庭主婦/家庭主夫）

□ 其他：＿＿＿＿＿＿＿＿＿＿

7. 你的職業：＿＿＿＿＿＿＿＿＿＿
8. 請填寫每位同住家庭成員的姓名（請用簡稱）、性別及每位家庭成員與你的關係。

| 簡稱（同住家庭成員） | 性別 | 與受訪者的關係（請參考關係代碼） |
| --- | --- | --- |
|  | □ 男 □ 女 |  |
|  | □ 男 □ 女 |  |
|  | □ 男 □ 女 |  |
|  | □ 男 □ 女 |  |

**關係代碼**

| A. 配偶 | G. 父母/主要照顧者 | M. 同父異母的兄弟姊妹 |
| --- | --- | --- |
| B. 同居 | H. 繼父母 | N. 養兄弟姊妹 |
| C. 子/女（包括領養） | I. 養父母 | O. 配偶之兄弟姊妹 |
| D. 繼子/女 | J. 配偶之父母 | P. 孫兒/女 |
| E. 養子/女 | K. 兄弟姊妹（包括領養） | Q. 祖父母 |
| F. 女婿/媳婦 | L. 繼兄弟姊妹 | R. 其他（請註明） |

9. 請填寫家庭中同住孩子的資料（年齡、性別、關係）。

1. 男 / 女, 年齡 ＿＿＿＿ （關係：	）

2. 男 / 女, 年齡 ＿＿＿＿ （關係：	）

3. 男 / 女, 年齡 ＿＿＿＿ （關係：	）

10. 請填寫孩子不同住的兄弟姊妹資料（年齡、性別、關係）。

1. 男 / 女, 年齡 ＿＿＿＿ （關係：	）

2. 男 / 女, 年齡 ＿＿＿＿ （關係：	）

3. 男 / 女, 年齡 ＿＿＿＿ （關係：	）

11. 每月家庭總收入：

□ 沒有收入

□ 領取綜援

□ 低於港幣10,000

□ 港幣10,000 - 19,999

□ 港幣20,000 - 24,999

□ 港幣25,000 - 29,999

□ 港幣30,000 - 39,999

□ 港幣40,000 - 59,999

□ 港幣60,000 - 79,999

□ 港幣80,000或以上

12. 你這家的房屋類型屬於？

□ 公共房屋單位

□ 私人住宅單位

□ 其他：＿＿＿＿＿＿＿＿＿＿

家中的實用面積:＿＿＿＿呎

13. 家庭是否正接受任何政府補助/援助（可複選）：

□ 沒有接受任何政府補助/援助

□ 綜合社會保障援助（綜援）

□ 學校書簿津貼計劃

□ 在職家庭津貼（職津）

□ 學費減免計劃

□ 其他：＿＿＿＿＿＿＿＿＿＿

14. 你的家庭是否面臨任何經濟困難：

□ 是

□ 否

15. 參加BBJC2服務計劃的日期：＿＿＿＿＿＿＿＿＿＿

# 第二部份：目標孩子個人資料

第二部分為孩子的基本個人資料，包括孩子的身心情況。

1. 孩子的性別：

□ 男性

□ 女性

□ 其他

2. 孩子的年齡：＿＿＿＿＿＿＿＿＿＿
3. 孩子的種族/族裔：

□ 華人

□ 印尼

□ 菲律賓

□ 白人

□ 印度

□ 巴基斯坦

□ 尼泊爾

□ 日本

□ 泰國

□ 其他: 請註明：＿＿＿＿＿＿＿＿＿＿

4. 誰是主要照顧者：

□ 父親

□ 母親

□ 繼父

□ 繼母

□ 兄弟姐妹

□ 祖父/外公

□ 祖母/外婆

□ 親戚

□ 其他：＿＿＿＿＿＿＿＿＿

5. 孩子每天可享用的餐種類 (可複選)：

□ 早餐

□ 午餐

□ 晚餐

□ 零食（	）次

□ 並無限制

6. 孩子正餐的食物類型（可複選）：

□ 新鮮烹煮、營養均衡的正餐（例如：米飯、麵條、蔬菜、新鮮肉類、水果）

□ 高度加工食品（例如：穀類食品、罐頭肉、香腸、火腿、即食麵或杯麵）

□ 快餐

□ 零食

□ 其他：＿＿＿＿＿＿＿＿＿

7. 孩子每天的睡眠時間（包含小睡）：

□ 少於 8 小時

□ 8–10 小時

□ 多於 10 小時

8. 孩子的體重：

□ <20kg

□ 20-30kg

□ 31-40kg

□ 41-50kg

□ >50kg

9. 孩子的身高：

□ <100cm

□ 100-110cm

□ 111-120cm

□ 121-130cm

□ 131-140cm

□ 141-150cm

□ >150cm

10. 孩子是否有確診的特殊教育需要/心理或精神健康問題 ?

(例如：智力和發展障礙、自閉症譜系障礙、注意力不足或過度活躍、讀寫障礙)

□ 否

□ 是

特殊教育需要/心理或精神健康問題紀錄:_________________

確診單位/機構: _________________

確診年份: _________________

支援服務: _______________________________ □ 不適用

藥物治療: _________________ □ 不適用 (是否定期服用:是□，否□ )

特殊學校就讀情況：是□，否□

11. 孩子有沒有因懷疑被傷害/虐待而曾召開「保護懷疑受虐待兒童多專業個案會議(MDCC)」?

□ 有，請簡述發生了什麼事情：__________________________

□ 否

□ 不知道

# 第三部份：家長背景資料

第三部分涉及家長的背景資料。若該兒童的非在囚家長同時為主要照顧者，部分問題將與第一部分的問題重複。

1. 在囚家長的性別：

□ 男性

□ 女性

□ 其他

2. 非在囚家長的性別：（如果是照顧者本人便不用填寫）

□ 男性

□ 女性

□ 其他

□ 不適用 （例如：單親家庭）

3. 在囚家長與非在囚家長的種族/族裔：

在囚家長：

□ 華人

□ 印尼

□ 菲律賓

□ 白人

□ 印度

□ 巴基斯坦

□ 尼泊爾

□ 日本

□ 泰國

□ 其他: 請註明：＿＿＿＿＿＿＿＿＿＿

非在囚家長：（如果是照顧者本人便不用填寫）

□ 華人

□ 印尼

□ 菲律賓

□ 白人

□ 印度

□ 巴基斯坦

□ 尼泊爾

□ 日本

□ 泰國

□ 其他: 請註明：＿＿＿＿＿＿＿＿＿＿

4. 在囚家長的婚姻狀況：

□ 已婚/再婚

□ 離婚/分居

□ 同居

□ 喪偶

□ 其他：＿＿＿＿＿＿＿＿＿

5. 非在囚家長的婚姻狀況：

□ 已婚/再婚

□ 離婚/分居

□ 同居

□ 喪偶

□ 其他：＿＿＿＿＿＿＿＿＿

6. 在囚家長的教育程度：

□ 小學或以下

□ 初中

□ 高中

□ 大專（副學士/文憑/證書）

□ 大學（學士學位）

□ 大學以上

7. 非在囚家長的教育程度：（如果是照顧者本人便不用填寫）

□ 小學或以下

□ 初中

□ 高中

□ 大專（副學士/文憑/證書）

□ 大學（學士學位）

□ 大學以上

8. 非在囚家長的就業狀況：（如果是照顧者本人便不用填寫）

□ 全職

□ 兼職

□ 自由職業者

□ 待業

□ 退休

□ 持家者（家庭主婦/家庭主夫）

□ 其他：＿＿＿＿＿＿＿＿＿

9. 非在囚家長職業：＿＿＿＿＿＿＿＿＿

# 第四部份：同住/直系家庭成員精神健康歷史

第四部分為同住或直系家庭成員精神健康歷史。

1. 同住家庭成員有沒有使用成癮性物質/濫用藥物？

□ 有，成員：＿＿＿＿＿＿＿＿＿，成癮性物質：＿＿＿＿＿＿＿＿＿

□ 沒有

□ 不知道

2. 孩子直系或同住家庭成員曾有心理健康問題或精神障礙？

□ 有，直系/居家成員：＿＿＿＿＿＿＿＿＿，病歷：＿＿＿＿＿＿＿＿＿

□ 沒有

□ 不知道

3. 家中是否有人在接受藥物治療？

□ 是，居家成員：＿＿＿＿＿＿＿＿＿，服用藥物/病歷：＿＿＿＿＿＿＿＿＿

□ 否

□ 不知道

# 第五部份：家庭成員監禁歷史

第五部分為家庭成員的監禁歷史。

1. 非在囚家長有沒有違法紀錄？

□ 有，違法紀錄： ＿＿＿＿＿＿＿＿＿

□ 沒有

2. 在囚家長是否有涉及虐兒的事件或紀錄？

□ 是，請具體說明 (例如事發日期、案情簡介、是否有被調查/定罪或判決情況、是否有持續的社工跟進或法庭監管令等)：＿＿＿＿＿＿＿＿＿＿＿＿＿＿＿＿＿

□ 否

3. 非在囚家長是否有涉及虐兒的事件或紀錄？

□ 是，請具體說明 (例如事發日期、案情簡介、是否有被調查/定罪或判決情況、是否有持續的社工跟進或法庭監管令等)：＿＿＿＿＿＿＿＿＿＿＿＿＿＿＿＿＿

□ 否

4. 在囚家長的控罪：＿＿＿＿＿＿＿＿＿
5. 刑期（數字）：＿＿＿＿＿＿＿＿＿
6. 入獄次數：＿＿＿＿＿＿＿＿＿
7. 孩子是否有探望在囚家長？

□ 是, 多久探訪一次：＿＿＿＿＿＿＿＿＿＿

□ 否

8. 家長被囚禁期間，孩子有沒有獲得第三方的經濟支援？（例如親戚援助）

□ 是

□ 否

# 第六部份：社工家訪觀察

第六部分為社工家訪觀察。請根據家訪觀察，逐項勾選「是」或「否」，並在有需要的地方填寫備註，特別是關於孩子與他人共用床位、住處安全性及孩子健康狀況等問題。

|  | 是 | 否 | 備註 |
| --- | --- | --- | --- |
| 1. 住處看起來安全。 | □ | □ |  |
| 2. 住處大致乾淨。 | □ | □ |  |
| 3. 住處光線充足。 | □ | □ |  |
| 4. 住處有基本設施，供孩子睡覺、玩耍、學習和休息。 | □ | □ |  |
| 5. 孩子與其他人（父母、異性等）共用一張床。 | □ | □ | 若是, 請說明與誰以及年齡差距：＿＿＿＿＿＿＿＿＿＿＿＿ |
| 6. 住處為孩子提供個人空間（如床位、書櫃、衣櫃）。 | □ | □ |  |
| 7. 住處有烹飪設備，並確實用於烹煮孩子與家人所需的膳食。 | □ | □ |  |
| 8. 從街道到住處的通道光線充足且暢通。 | □ | □ |  |
| 9. 社區環境有一些兒童遊樂或休閒設施。 | □ | □ |  |
| 10. 孩子穿著乾淨且合身的衣物。 | □ | □ |  |
| 11. 孩子有明顯的傷痕或傷口。 | □ | □ |  |
| 12. 住處經常有非親屬或孩子不熟悉的成年人出入。 | □ | □ |  |

# 第七部份：檢核表（家長/主要照顧者版本）

**填答說明**

第七部份分為兩部分：

**第一部分**：請參考過去兩個月的狀況作答

這部分主要針對孩子近期的日常生活、情緒行為和社交狀況。

**第二部分**：請根據孩子自出生起至目前為止的所有經驗作答

這部分包含較為敏感的題目（如身體或心理虐待、性侵犯、疏忽照顧、早期犯罪徵兆等）。回答時若有任何不舒服，請通知我, 我看情況再處理。

__________

第一部分：請依照在過去兩個月的經驗回答。請在「是」或「否」之間勾選，若無符合情況請勾選「否」。**如有需要，請填寫備註。請注意，本部分中「經常」指一週七天中有三天或以上。**

1. **生理範疇**

|  |  | 是 | 否 | 備註 |
| --- | --- | --- | --- | --- |
| 1.1. 個人衛生 | a. 孩子定期洗頭（至少隔日）。 | □ | □ |  |
|  | b. 孩子定期刷牙（每天 2 次）。 | □ | □ |  |
| 1.2. 睡眠 | a. 孩子經常拒絕上床睡覺。 | □ | □ |  |
|  | b. 孩子經常難以入睡。 | □ | □ |  |
|  | c. 孩子經常睡得太久。 | □ | □ |  |
|  | d. 孩子常常小睡。 | □ | □ |  |
|  | e. 孩子在睡覺時經常尿床。 | □ | □ |  |
| 1.3. 營養 | a. 孩子都有定時享用正餐。 | □ | □ |  |
|  | b. 孩子吃得很少。 | □ | □ |  |
|  | c. 孩子常以零食取代正餐。 | □ | □ |  |
|  | d. 孩子有挑食的習慣。 | □ | □ |  |

2. **心理範疇**

|  |  | 是 | 否 | 備註 |
| --- | --- | --- | --- | --- |
| 2.1. 情緒 | a. 孩子總是煩躁/悲傷/缺乏動力和興趣。 | □ | □ |  |
|  | b. 孩子總是顯得擔憂/缺乏安全感。 | □ | □ |  |
| 2.2. 創傷後壓力症(PTSD) | a. 孩子會迴避任何引起創傷回憶的人、地點或活動。 | □ | □ |  |
|  | b. 孩子經常發惡夢或有不愉快的夢境。 | □ | □ |  |
|  | c. 孩子變得容易受到驚嚇（例如有人從背後靠近時會嚇一跳）。 | □ | □ |  |
| 2.3. 依附關係 | a. 孩子變得越來越黏我。 | □ | □ |  |
|  | b. 孩子願意與我坦誠分享任何事。 | □ | □ |  |
|  | c. 孩子刻意避免與我親近。 | □ | □ |  |
| 2.4. 遮瞞與坦白 | a. 我一直避免提及在囚家長的缺席。 | □ | □ |  |
|  | b. 我會編造理由（如工作、旅行）來解釋在囚家長的離開。 | □ | □ |  |
|  | c. 孩子曾目睹該家長被逮捕。 | □ | □ |  |
|  | d. 我有向孩子解釋過，他/她的家長正在服刑。 | □ | □ |  |
| 2.5. 與在囚家長的聯繫 | a. 孩子知道家長何時會釋放。 | □ | □ |  |
|  | b. 我讓孩子自己決定何時探望在囚家長。 | □ | □ |  |
|  | c. 在囚家長與孩子定期以不同方式（如信件、探視、電話）保持聯繫。 | □ | □ |  |
|  | d. 孩子曾表示因家長被囚而遭到他人排斥（同學、鄰居等）。 | □ | □ |  |
| 2.6. 自殺念頭/自殘行為 | a. 孩子曾嘗試故意傷害自己。 | □ | □ |  |
|  | b. 孩子曾做過想結束自己生命的行為。 | □ | □ |  |
|  | c. 孩子曾表示希望自己死去或一睡不醒。 | □ | □ |  |

3. **社交範疇**

|  |  | 是 | 否 | 備註 |
| --- | --- | --- | --- | --- |
| 3.1. 社交功能 | a. 孩子和同齡朋友相處良好。 | □ | □ |  |
|  | b. 孩子曾在學校受到欺凌。 | □ | □ |  |
|  | c. 孩子目前有戀愛關係。 | □ | □ |  |
|  | d. 孩子曾參與童黨或幫派活動。 | □ | □ |  |
|  | e. 孩子沉迷於遊戲，妨礙日常作息。 | □ | □ |  |
| 3.2. 學校生活功能 | a. 孩子幾乎跟不上課業進度。 | □ | □ |  |
|  | b. 孩子能連續五天都去上學。 | □ | □ |  |
|  | c. 孩子經常不願意上學。 | □ | □ |  |
|  | d. 孩子經常被老師投訴。 | □ | □ |  |
|  | e. 孩子常與其他兒童或同學打架。 | □ | □ |  |
|  | f. 孩子有偷竊的行為。 | □ | □ |  |
| 若該孩子有兄弟姐妹，請完成3.3以下部分 | | | | |
| 3.3. 兄弟姐妹關係 | a. 孩子與兄弟姐妹相處融洽。 | □ | □ |  |
|  | b. 孩子與兄弟姐妹常有嚴重衝突。 | □ | □ |  |

4. **家長職能**

|  | 是 | 否 | 備註 |
| --- | --- | --- | --- |
| a. 我覺得很難追蹤孩子每天的行程。 | □ | □ |  |
| b. 孩子經常在家卻沒有成人照顧。 | □ | □ |  |
| c. 孩子主要的照顧者經常更換。 | □ | □ |  |

第二部分：**以下問題為了更全面了解孩子的成長經驗，可能較為敏感，回答時若有任何不舒服適，請隨時告訴我。**請回想自孩子出生以來（或自孩子尚在您的照顧範範圍內），是否曾經出現以下情況：

5. **與虐待/疏忽照顧有關**

|  |  | 是 | 否 | 備註 |
| --- | --- | --- | --- | --- |
| 5.1. 身體虐待 | a. 家人曾因孩子犯錯/無故而對孩子使用體罰。 | | | |
|  | □ 是 ，包含下列情況：  □ 用手掌打孩子臀部  □ 用物件打孩子臀部  □ 拍打孩子手、臂、腿或其他部位  □ 用物件打孩子手、臂、腿或其他部位  □ 用物件打孩子頭部或臉部  □ 捏孩子  □ 搖晃孩子  □ 其他：＿＿＿＿＿＿＿ | | □ |  |
| 5.2. 心理虐待 | a. 家人經常對孩子說帶有侮辱或傷害性的話。 | □ | □ |  |
|  | b. 我感覺家中有人討厭孩子。 | □ | □ |  |
|  | c. 我曾想過「如果孩子沒出生就好了」。 | □ | □ |  |
|  | d. 我常跟孩子說，他/她遺傳了被囚家長的負面特質。 | □ | □ |  |
| 5.3. 性侵犯 | a. 我相信孩子曾遭性侵犯或不當性接觸。 | | | |
|  | □ 是, 包含下列情況：  □ 有人曾以性方式觸碰孩子，或讓孩子觸碰他們。  □ 有人曾要求孩子做或觀看色情行為。  □ 孩子曾實際遭到性侵犯。  □ 有人以傷害或誣衊相威脅，逼迫孩子進行性行為。  □ 有人曾對孩子講黃色笑話。  □ 孩子曾被迫觀看色情影片或圖片。  □ 其他：＿＿＿＿＿＿＿ | | □ |  |
| 5.4. 疏忽照顧(情感) | a. 孩子很少感受到家人的關愛。 | □ | □ |  |
|  | b. 家人從未把孩子當作真正的家庭成員。 | □ | □ |  |
| 5.5. 疏忽照顧(身體) | a. 孩子經常沒有人照顧，也無人確保其安全。 | □ | □ |  |
|  | b. 孩子缺乏足夠的食物或基本生活/衛生照料。 | □ | □ |  |

6. **與早期青少年犯罪有關**

|  | 是 | 否 | 備註 |
| --- | --- | --- | --- |
| a. 孩子沮喪時會亂發脾氣。 | □ | □ |  |
| b. 孩子沮喪時會破壞物品。 | □ | □ |  |
| c. 孩子沮喪時會攻擊他人。 | □ 是，他/她會：  □ 用手攻擊他人  □ 使用武器攻擊  □ 其他：＿＿＿＿＿ | □ |  |

__________

**家長/主要照顧者版本結束。若問卷中某些題目讓您感到不舒服，請告知社工或工作人員，我們會與您進一步討論和提供協助。**

# 第八部份：檢核表（兒童版本）

**填答說明**

第八部份分為兩部分：

**第一部分**：請參考過去兩個月的狀況作答

這部分主要想了解您**最近**在學校與家中生活的情況。

**第二部分**：請依照從小到大的經驗的經驗作答

這部分包含較為敏感的成長經驗（如身體/心理虐待、性侵犯、疏忽照顧等），回答時若有任何不舒服，請通知我,我看情況再處理。

__________

**第一部分：請依照在過去兩個月的經驗回答。請在「是」或「否」之間勾選，若無符合情況請勾選「否」。如有需要，請填寫備註。請注意，本部分中「經常」指一週七天中有三天或以上。**

1. **生理範疇**

|  |  | 是 | 否 | 備註 |
| --- | --- | --- | --- | --- |
| 1.1. 個人衛生 | a. 我會定期洗頭（至少隔日）。 | □ | □ |  |
|  | b. 我會定期刷牙（每天2次）。 | □ | □ |  |
| 1.2. 睡眠 | a. 我常常想要晚一點才睡。 | □ | □ |  |
|  | b. 我常常難以入睡。 | □ | □ |  |
|  | c. 我睡得很多。 | □ | □ |  |
|  | d. 我喜歡常常小睡。 | □ | □ |  |
|  | e. 我經常尿床。 | □ | □ |  |
| 1.3. 營養 | a. 我每天有2-3次正餐。 | □ | □ |  |
|  | b. 我的正餐有肉、蔬菜和米飯。 | □ | □ |  |
|  | c. 我不愛正餐，因為不好吃，所以吃得不多。 | □ | □ |  |

2. **心理範疇**

|  |  | 是 | 否 | 備註 |
| --- | --- | --- | --- | --- |
| 2.1. 情緒 | a. 我幾乎每天都感到煩躁/悲傷/沒心情做事。 | □ | □ |  |
|  | b. 我幾乎每天都在擔心之後會發生什麼。 | □是,擔心：  ＿＿＿＿ | □ |  |
|  | c. 我覺得自己一無是處。 | □ | □ |  |
|  | d. 我感到絕望。 | □ | □ |  |
|  | e. 我感到羞恥。 | □ | □ |  |
| 2.2. 創傷後壓力症(PTSD) | a. 我會避開讓我想起不好回憶的地方。 | □ | □ |  |
|  | b. 我經常發惡夢或有不愉快的夢境。 | □ | □ |  |
|  | c. 我變得非常容易受到驚嚇或突然被嚇到（例如有人突然靠近我）。 | □ | □ |  |
| 2.3. 依附關係/信任 | a. 我變得越來越黏主要照顧者。 | □ | □ |  |
|  | b. 我可以跟照顧者分享任何事。 | □ | □ |  |
|  | c. 我盡量不和照顧者走得太近。 | □ | □ |  |
|  | d. 我不想跟照顧者合作。 | □ | □ |  |
|  | e. 我無法信任家裡的任何人。 | □ | □ |  |
|  | f. 我從來沒有被問過意見，也沒有人關心我在想什麼。 | □ | □ |  |
| 2.4. 遮瞞與坦白 | a. 你知道你的（在囚家長）現在在哪裡嗎？ | | | |
|  | ☐ 知道，我的家長在 ＿＿＿＿＿。  你是怎樣知道的？ ☐ 有人告訴我（例如：＿＿＿＿＿＿＿＿＿＿） ☐ 自己猜的 ☐ 其他方式：＿＿＿＿＿＿ | | ☐ 不知道 |  |
|  | b. （知道家長被囚禁的兒童才回答以下問題)   如果你知道家長**被囚禁**，以下說法你是否同意？ | | | |
|  | i. 我感覺家裡的人都不願意談論他/她。 | □ | □ |  |
|  | ii. 我相信家長被監禁的理由。 | □ | □ |  |
|  | iii. 我知道他/她什麼時候會出來。 | □ | □ |  |
|  | iv. 我曾經親眼看見家長被逮捕。 | □ | □ |  |
| 2.5. 與在囚家長的聯繫 | a. （知道家長被囚禁的兒童才回答以下問題)   我有去探望被囚禁的家長。 | | | |
|  | □ 有，探訪頻率：  □ 每星期一次  □ 每兩星期一次  □ 每月一次  □ 每三個月一次  □ 每半年一次  □ 每年一次或更少  是我自己決定的嗎？  □ 是  □ 不是 | | □ 沒有 |  |
|  | b. 我透過信件或電話等方式和被囚禁的家長保持聯繫。 | □ | □ |  |
| 2.6. 自殺念頭/自殘行為 | a. 我曾經希望自己死掉，或睡了就不要再醒來。 | □ | □ |  |
|  | b. 我曾故意傷害自己。 | □ | □ |  |
|  | c. 我曾想結束自己的生命。 | □ | □ |  |

3. **社交範疇**

|  |  | 是 | 否 | 備註 |
| --- | --- | --- | --- | --- |
| 3.1. 社交功能 | a. 我幾乎和其他孩子相處不來。 | □ | □ |  |
|  | b. 我經常被同學欺負。 | □ | □ |  |
|  | c. 我有男朋友或女朋友。 | □ | □ |  |
|  | d. 我大多時間都在街上跟朋友混在一起。 | □ | □ |  |
|  | e. 我每天花很多時間打遊戲機。 | □ | □ |  |
| 3.2. 學校生活功能 | a. 我幾乎跟不上學校的課業。 | □ | □ |  |
|  | b. 我常常不想上學。 | □ | □ |  |
|  | c. 我常常被老師罵或投訴。 | □ | □ |  |
|  | d. 我常常跟同學打架。 | □ | □ |  |
|  | e. 我有偷竊的行為。 | □ | □ |  |
| 若該孩子有兄弟姐妹，請完成3.3以下部分 | | | | |
| 3.3. 兄弟姐妹關係 | a. 我跟兄弟姐妹相處融洽。 | □ | □ |  |
|  | b. 我跟兄弟姐妹常有嚴重衝突。 | □ | □ |  |

4. **家長職能**

|  | 是 | 否 | 備註 |
| --- | --- | --- | --- |
| a. 我的照顧者常常忘記我在哪裡、在做什麼。 | □ | □ |  |
| b. 我時常一個人在家，沒有任何大人。 | □ | □ |  |
| c. 最近我換了好幾位照顧者。 | □ | □ |  |

第二部分：**以下問題為了更全面了解你的成長經驗，可能較為敏感，回答時若有任何不舒服，請隨時告訴我。**請依照自有記憶以來的經驗作答：

5. **與虐待/疏忽照顧有關**

|  |  | 是 | 否 | 備註 |
| --- | --- | --- | --- | --- |
| 5.1. 身體虐待 | a. 過去兩個月，我因為做錯事或令人不滿意或者沒有理由而被照顧者或家人打。 | | | |
|  | □ 是，對方曾經：  □ 用手掌打我屁股  □ 用物件打我屁股  □ 用手掌打我手、手臂、腿或身體其他部位  □ 用物件打我手、手臂、腿或身體其他部位  □ 用物件打我頭部或臉部  □ 捏我  □ 搖晃我  □ 其他：＿＿＿＿＿＿＿ | | □ |  |
| 5.2. 心理虐待 | a. 家裡的人經常對我說侮辱或傷害性的話（例如「笨」「懶」「醜」）。 | □ | □ |  |
|  | b. 我一直感覺家裡有人討厭我。 | □ | □ |  |
|  | c. 有人對我說過「沒人想要你」。 | □ | □ |  |
|  | d. 我經常被拿來和入獄/不在家居住的家長做負面比較。 | □ | □ |  |
| 5.3. 性侵犯 | a. 我覺得/懷疑自己曾遭到性侵犯或被迫做與性有關的事。 | | | |
|  | □ 是，包含下列情況：  □ 有人曾碰我私人部位，或想讓我碰他們的私人部位。  □ 有人想要我做或觀看與性有關的事情。  □ 有人性侵犯我。  □ 有人威脅要傷害或誣衊我，除非我跟他/她做某些性行為。  □ 有人強迫我和他/她進行性行為。  □ 有人跟我說過黃色笑話，我並不想聽。  □ 我曾被迫觀看色情影片或圖片。  □ 其他：＿＿＿＿＿＿＿ | | □ |  |
| 5.4. 疏忽照顧(情感) | a. 我從來沒有感受到被愛。 | □ | □ |  |
|  | b. 我覺得自己不算是家裡的一份子。 | □ | □ |  |
| 5.5. 疏忽照顧(身體) | a. 我的照顧者有好好給我食物、衣服、醫療等基本需求。 | □ | □ |  |
|  | b. 當我需要時，我不確定有沒有人會來保護我。 | □ | □ |  |

6. **與早期青少年犯罪有關**

|  | 是 | 否 | 備註 |
| --- | --- | --- | --- |
| a. 我在沮喪時會發脾氣。 | □ | □ |  |
| b. 我在沮喪時會破壞東西。 | □ | □ |  |
| c. 我在沮喪時會打人。 | □是, 因為:  ___________________ | □ |  |

7. **污名感知**

|  | 是 | 否 | 備註 |
| --- | --- | --- | --- |
| a. 我曾覺得自己被某些權威/師長看輕或取笑。 | □ | □ |  |
| b. 我曾被同學、朋友、鄰居、親戚或專業人士排斥。 | □是, 因為:  ___________________ | □ |  |

__________

**兒童版本結束。若你在作答時感到不舒服，或需要進一步討論，**

**請告知社工或工作人員，我們會與您進一步討論和提供協助。**
