# Supplementary material for: Development and pilot testing of a risk assessment checklist for children of incarcerated parents in Hong Kong
Source: Front Sociol. 2026 Sep 24;11:1936866. doi: 10.3389/fsoc.2026.1936866 (PMC13647460; doi:10.3389/fsoc.2026.1936866)
Supplement: Supplementary file 2 [file Supplementary_file_2.DOCX]

Supplementary Material

This file contains the English version of the Risk Assessment Checklist, with a total of 24 pages.

**Blue Bus Jockey Club Together We Grow Project – Child Risk Assessment Checklist (Aged 6-11)**

Social Worker’s Name: _____________ Case Number: _____________ Date: _____________

Case Status: New case / Old case (please circle)

1. **Purpose and Function of this Checklist**
   1.1. This checklist is a risk assessment tool that aims to help social workers systematically identify the emotional, psychological, and environmental difficulties that children (aged 6 to 11) may face.
   1.2. Function of Risks Assessed
        1.2.1. To summarize the child’s level of risk in emotional, developmental, behavioral, physical, and cultural domains.
        1.2.2. To provide social workers and caregivers with concrete and feasible recommendations, such as referral for consultation or increased school support.
2. **Checklist Administration, Versions, and Sections**
   2.1. The Checklist has two versions: a parent/primary caregiver version and a child version. The social worker reads the questions aloud and completes the form based on the respondent’s answers.
   2.2. The two versions are divided into sections as follows:

|  | **Parent/Primary Caregiver Version** | **Child Version** |
| --- | --- | --- |
| 1 | Caregiver Demographic Information |  |
| 2 | Child Personal Information |  |
| 3 | Parent Background Information |  |
| 4 | Family History of Mental Health |  |
| 5 | Family History of Incarceration |  |
| 6 | Social Worker Home Observation |  |
| 7 | Child Risk Assessment (Parent/Primary Caregiver Version) |  |
| 8 |  | Child Risk Assessment (Child Version) |

3. **Features of the Checklist**
   3.1. Grounded in theory and both Chinese and international literature.
   3.2. Culturally and contextually adapted.
        3.2.1. Ensures the use of culturally appropriate language.
        3.2.2. Takes into account social norms and values related to family and incarceration.
        3.2.3. Adjusts questions according to the local context.
   3.3. Simple and easy to understand, tailored to the characteristics of the respondents (age and educational level).
   3.4. Wording is careful to avoid triggering emotional distress in respondents.
4. **Administration Instructions for Social Workers**
   4.1. It is recommended that the parent/primary caregiver and child versions be administered separately to protect the privacy and authenticity of both parties.
   4.2. The parent/primary caregiver must have been in the role of caring for the child for at least six months.
   4.3. Ensure that the interview setting is quiet, comfortable, and free from distractions.
   4.4. It is recommended that the questionnaire be completed during a home visit to facilitate observation of the home environment.
   4.5. The questionnaire is expected to take approximately 1 to 2 hours to complete. It may be completed over several sessions within a period of time, but it is recommended to complete it as early as possible (e.g., within the first month of the case).
   4.6. The wording of the child version has been adjusted to suit the comprehension level of children aged 6 to 11. If a child has difficulty understanding a particular word or sentence, the social worker may explain as appropriate.
5. **Introduction and Informed Participation**
   5.1. You may use the following statement for the introduction: “This Checklist aims to help us better understand you and your child’s situation so that we can provide more tailored support.
   5.2. Emphasize that participants have the right to skip any question and may also request that certain answers not be recorded. Whether the parent/primary caregiver participates will not affect the support services they receive.
6. **Confidentiality and Information Disclosure**
   6.1. Unless a potential risk is identified under the Mandatory Reporting of Child Abuse Ordinance, effective January 2026, the social worker will not actively disclose answers to anyone.
   6.2. If a parent or child requests to know the other party’s answers, the social worker will use discretion and handle the request appropriately.
   6.3. If a parent requests access to the child’s information under the Personal Data (Privacy) Ordinance, such requests will be handled in accordance with relevant regulations.
7. Handling of Sensitive Information and High-Risk Situations
   7.1. If any situation involving crisis, harm, or abuse is identified, please immediately follow the relevant legal requirements or the organization's child protection procedures.

**Parent/Primary Caregiver Questionnaire**

Please assist the parent or primary caregiver in completing this questionnaire for the target child ______. The questionnaire is divided into five sections: Caregiver Demographic Information, Child's Personal Information, Parent Background Information, Family History of Mental Health, and Family History of Incarceration. Please ensure the accuracy of the answers and explain the content of the questions as needed.

# Section 1: Caregiver Basic Information

Section 1 covers the primary caregiver's basic personal background information. To ensure the representativeness of the information, the primary caregiver must have been in the role of caring for the child for at least six months.

1. You are the child's:
   □ Father
   □ Mother
   □ Grandfather (Paternal/Maternal)
   □ Grandmother (Paternal/Maternal)
   □ Other relationship (please specify): ＿＿＿＿＿＿＿＿＿
2. Gender:
   □ Male
   □ Female
   □ Other
3. Age: ＿＿＿＿＿＿＿＿＿＿
4. Ethnicity:
   □ Chinese
   □ Indonesian
   □ Filipino
   □ White
   □ Indian
   □ Pakistani
   □ Nepalese
   □ Japanese
   □ Thai
   □ Other: Please specify: ＿＿＿＿＿＿＿＿＿＿
5. Education Level:
   □ Primary school or below
   □ Junior secondary
   □ Senior secondary
   □ Post-secondary (Associate Degree/Diploma/Certificate)
   □ University (Bachelor's Degree)
   □ Postgraduate
6. Employment Status:
   □ Full-time
   □ Part-time
   □ Freelancer

□ Unemployed
□ Retired
□ Homemaker (Housewife/husband)

□ Other: ＿＿＿＿＿＿＿＿＿＿

7. Your occupation: ＿＿＿＿＿＿＿＿＿＿
8. Please list the abbreviated name of the co-residing family member, sex, and relationship to the respondent.

| Abbreviated Name (co-residing family member) | Gender | Relationship to respondent (use relationship codes) |
| --- | --- | --- |
|  | □ Male □ Female |  |
|  | □ Male □ Female |  |
|  | □ Male □ Female |  |
|  | □ Male □ Female |  |

**Relationship Codes**

| A. Spouse | G. Parent/Guardian | M. Half-sibling |
| --- | --- | --- |
| B. Cohabitant | H. Stepparent | N. Adoptive sibling |
| C. Son/daughter (including adopted) | I. Adoptive parent | O. Spouse’s sibling |
| D. Stepchild | J. Parent-in-law | P. Grandchild |
| E. Adopted child | K. Sibling (including adopted) | Q. Grandparent |
| F. Son-in-law/daughter-in-law | L. Stepsibling | R. Other (please specify) |

9. Please provide details of the co-residing children in the household (age, gender, relationship).
1. Male / Female, Age ＿＿＿＿ (Relationship: )
2. Male / Female, Age ＿＿＿＿ (Relationship: )
3. Male / Female, Age ＿＿＿＿ (Relationship: )
10. Please provide details of the child’s non-co-residing siblings (age, gender, relationship).
a. Male / Female, Age ＿＿＿＿ (Relationship: )
b. Male / Female, Age ＿＿＿＿ (Relationship: )
c. Male / Female, Age ＿＿＿＿ (Relationship: )
11. Total monthly household income:

□ No income
□ Receiving Comprehensive Social Security Assistance (CSSA)
□ Below HKD 10,000
□ HKD 10,000 - 19,999
□ HKD 20,000 - 24,999
□ HKD 25,000 - 29,999
□ HKD 30,000 - 39,999
□ HKD 40,000 - 59,999
□ HKD 60,000 - 79,999
□ HKD 80,000 or above

12. What is your housing type?
    □ Public Housing Unit
    □ Private Residential Unit
    □ Other: _____________
    Usable floor area of home: _______ sq. ft.
13. Is the household receiving any government subsidies/assistance? (Multiple selections possible):
    □ Not receiving any government subsidies/assistance
    □ Comprehensive Social Security Assistance (CSSA)
    □ School Textbook Assistance Scheme
    □ Working Family Allowance
    □ Tuition Fee Reduction Scheme
    □ Other: _____________
14. Is your household experiencing any financial difficulties?
    □ Yes
    □ No
15. Date of joining the BBJC2 service program: _____________

# Section 2: Child’s Personal Details

Section 2 covers the child's basic personal information, including their physical and mental conditions.

1. Child’s Gender:

□ Male
□ Female
□ Other

2. Child’s Age: ＿＿＿＿＿＿＿＿＿＿
3. Child’s Ethnicity:

□ Chinese
□ Indonesian
□ Filipino
□ White
□ Indian
□ Pakistani
□ Nepalese
□ Japanese
□ Thai
□ Other: Please specify: _____________

4. Who is the primary caregiver?
   □ Father
   □ Mother
   □ Stepfather
   □ Stepmother
   □ Sibling(s)
   □ Grandfather (Paternal/Maternal)
   □ Grandmother (Paternal/Maternal)
   □ Relative(s)
   □ Other: _____________
5. Number of meals the child has daily:

□ Breakfast

□ Lunch

□ Dinner

□ Snacks (	) times

□ No restrictions

6. Type of food for the child's main meals (Multiple selections possible):
   □ Freshly cooked, nutritionally balanced meals (e.g., rice, noodles, vegetables, fresh meat, fruit)
   □ Highly processed foods (e.g., cereal, canned meat, sausages, ham, instant noodles/cup noodles)
   □ Fast food
   □ Snacks
   □ Other: _____________
7. Child's daily sleep duration (including naps):
   □ Less than 8 hours
   □ 8-10 hours
   □ More than 10 hours
8. Child's Weight:

□ <20kg
□ 20-30kg
□ 31-40kg

□ 41-50kg

□ >50kg

9. Child's Height:

□ <100 cm

□ 100–110 cm

□ 111–120 cm

□ 121–130 cm

□ 131–140 cm

□ 141–150 cm

□ >150 cm

10. Does the child have any diagnosed Special Educational Needs (SEN) or mental health conditions?
    (e.g., intellectual and developmental disabilities, Autism Spectrum Disorder, Attention Deficit Hyperactivity Disorder, dyslexia)

□ No

□ Yes

SEN / Mental Health Condition Record: _______________

Diagnosing Institution / Organization: _________________

Year of Diagnosis: _________________

Support Services: _______________________________ □ Not Applicable

Medication: ____________ □ Not applicable (If yes, is it taken regularly? □ Yes □ No)

Attends a special needs school: □ Yes □ No

11. Has a Multi-disciplinary Case Conference (MDCC) on Suspected Child Abuse ever been convened for the child?

□ Yes, please briefly describe what happened: __________________________

□ No

□ Don’t know

# Section 3: Parental Background Information

Section 3 covers the parents' background information. If the child's non-incarcerated parent is also the primary caregiver, some questions will overlap with those in Section 1.

1. Incarcerated parent's gender:
   □ Male
   □ Female
   □ Other
2. Non-incarcerated parent's gender: (Leave blank if this is the respondent/caregiver)
   □ Male
   □ Female
   □ Other
   □ Not Applicable (e.g., single-parent family)
3. Ethnicity of incarcerated and non-incarcerated parents:

Incarcerated Parent

□ Chinese
□ Indonesian
□ Filipino
□ White
□ Indian
□ Pakistani
□ Nepalese
□ Japanese
□ Thai
□ Other: Please specify: _____________

Non-incarcerated Parent: (Leave blank if this is the respondent/caregiver)

□ Chinese
□ Indonesian
□ Filipino
□ White
□ Indian
□ Pakistani
□ Nepalese
□ Japanese
□ Thai
□ Other: Please specify: _____________

4. Marital status of the child’s parent:

□ Single
□ Married / Remarried
□ Divorced / Separated
□ Cohabiting
□ Widowed
□ Other: _____________

5. Incarcerated parent’s education level:

□ Primary school or below
□ Junior secondary
□ Senior secondary
□ Post-secondary (Associate Degree/Diploma/Certificate)
□ University (Bachelor's Degree)
□ Postgraduate

6. Non-incarcerated parents’ employment status: (Leave blank if this is the respondent/caregiver)

□ Primary school or below
□ Junior secondary
□ Senior secondary
□ Post-secondary (Associate Degree/Diploma/Certificate)
□ University (Bachelor's Degree)
□ Postgraduate

7. Non-incarcerated parent’s employment status (Leave blank if this is the respondent/caregiver)

□ Full-time
□ Part-time
□ Freelancer

□ Unemployed
□ Retired
□ Homemaker (Housewife/husband)

□ Other: ＿＿＿＿＿＿＿＿＿＿

8. Non-incarcerated parent’s occupation: ＿＿＿＿＿＿＿＿＿

# Section 4: Co-residing / Immediate Family Member Mental Health History

Section 4 covers the mental health history of co-residing or immediate family members.

1. Do any co-residing family members use addictive substances or abuse drugs?
   □ Yes, Member: _____________, Substance: _____________,
   □ No
   □ Don't know
2. Have any immediate or co-residing family members of the child had mental health issues or psychiatric disorders?
   □ Yes, Immediate/Co-residing Member: _____________, Condition/History: __________
   □ No
   □ Don't know
3. Is anyone in the household currently taking medication?
   □ Yes, Household Member: _____________, Medication/Condition: _____________,
   □ No
   □ Don't know

# Section 5: Family Member Incarceration History

Section 5 covers the incarceration history of family members.

1. Does the non-incarcerated parent have any criminal record?
   □ Yes, Record: _____________
   □ No
2. Does the incarcerated parent have any history of child abuse incidents?
   □ Yes, please specify (e.g., date of incident, brief case description, investigation/conviction or sentencing status, ongoing social worker follow-up or court supervision order, etc.): __________________________
   □ No
3. Does the non-incarcerated parent have any history of child abuse incidents?
   □ Yes, please specify (e.g., date of incident, brief case description, investigation/conviction or sentencing status, ongoing social worker follow-up or court supervision order, etc.): __________________________
   □ No
4. Charges against the incarcerated parent: _____________
5. Sentence length (number): _____________
6. Number of times incarcerated: _____________
7. Does the child visit the incarcerated parent?
   □ Yes, how often: _____________
   □ No
8. Did the child receive financial support from a third party during the parent’s incarceration (e.g., assistance from relatives)?
   □ Yes
   □ No

# Section 6: Social Worker Home Visit Observations

Section 6 covers observations made by the social worker during home visits. Please tick "Yes" or "No" for each item based on home visit observations, and provide remarks where necessary, particularly regarding bed-sharing, home safety, and the child's health condition.

|  | Yes | No | Remarks |
| --- | --- | --- | --- |
| 1. The residence appears safe. | □ | □ |  |
| 2. The residence is generally clean. | □ | □ |  |
| 3. The residence has adequate lighting. | □ | □ |  |
| 4. The residence has basic facilities for the child to sleep, play, study, and rest. | □ | □ |  |
| 5. The child shares a bed with others (parents, opposite sex, etc.). | □ | □ | If yes, please specify with whom and the age difference：＿＿＿＿＿＿＿＿＿＿＿＿＿ |
| 6. The residence provides personal space for the child (e.g., bed, bookshelf, wardrobe). | □ | □ |  |
| 7. The residence has cooking facilities, which are used to prepare meals for the child and family. | □ | □ |  |
| 8. The access path from the street to the residence is well-lit and unobstructed. | □ | □ |  |
| 9. The community environment has some children's playground or leisure facilities. | □ | □ |  |
| 10. The child is wearing clean and well-fitting clothes. | □ | □ |  |
| 11. The child has visible bruises or wounds. | □ | □ |  |
| 12. Non-relatives or adults unfamiliar to the child frequently visit the residence. | □ | □ |  |

# Section 7: Checklist (Parent/Primary Caregiver Version)

**Instructions for Completion**

This section is divided into two parts:

**Part One**: Please answer based on the situation over the past two months

This part focuses on the child's recent daily life, emotional and behavioural state, and social functioning.

**Part Two**: Please answer based on all experiences the child has had from birth up to the present

This part contains more sensitive questions (e.g., physical or psychological abuse, sexual abuse, neglect, early signs of delinquency, etc.). If you feel uncomfortable while answering, please let me know, and I will handle the situation as appropriate.

__________

Part One: Please answer based on your experiences over the past two months. Please tick "Yes" or "No". If none of the situations apply, tick "No". Please provide remarks where necessary. Please note that in this section, "frequently" or “often” means three or more days out of seven.

1. **Physical Domain**

|  |  | Yes | No | Remarks |
| --- | --- | --- | --- | --- |
| 1.1. Personal Hygiene | a. The child washes their hair regularly (at least every other day). | □ | □ |  |
|  | b. The child brushes their teeth regularly (twice daily). | □ | □ |  |
| 1.2. Sleep | a. The child frequently refuses to go to bed. | □ | □ |  |
|  | b. The child frequently has difficulty falling asleep. | □ | □ |  |
|  | c. The child frequently sleeps for long periods. | □ | □ |  |
|  | d. The child frequently naps. | □ | □ |  |
|  | e. The child frequently wets the bed during sleep. | □ | □ |  |
| 1.3. Nutrition | a. The child has regular meals on time. | □ | □ |  |
|  | b. The child eats very little. | □ | □ |  |
|  | c. The child often replaces meals with snacks. | □ | □ |  |
|  | d. The child is a picky eater. | □ | □ |  |

2. **Psychological Domain**

|  |  | Yes | No | Remarks |
| --- | --- | --- | --- | --- |
| 2.1. Emotions | a. The child is always irritable / sad / lacks motivation and interest. | □ | □ |  |
|  | b. The child always appears worried. | □ | □ |  |
| 2.2. Post-traumatic Stress Disorder (PTSD) | a. The child avoids any people, places, or activities that trigger traumatic memories. | □ | □ |  |
|  | b. The child frequently has nightmares or unpleasant dreams. | □ | □ |  |
|  | c. The child has become easily startled (e.g., jumps when someone approaches from behind). | □ | □ |  |
| 2.3. Attachment | a. The child has become increasingly clingy towards me. | □ | □ |  |
|  | b. The child is willing to share anything with me openly and honestly. | □ | □ |  |
|  | c. The child deliberately avoids being close to me. | □ | □ |  |
| 2.4. Concealment and Disclosure | a. I have been avoiding mentioning the incarcerated parent's absence. | □ | □ |  |
|  | b. I make up reasons (e.g., work, travel) to explain the incarcerated parent's absence. | □ | □ |  |
|  | c. The child has witnessed the parent’s arrest. | □ | □ |  |
|  | d. I have explained to the child that his/her parent is serving a prison sentence. | □ | □ |  |
| 2.5. Contact with Incarcerated Parent | a. The child knows when the parent will be released. | □ | □ |  |
|  | b. I let the child decide when to visit the incarcerated parent. | □ | □ |  |
|  | c. The incarcerated parent and the child maintain regular contact through various means (e.g., letters, visits, phone calls). | □ | □ |  |
|  | d. The child has reported being rejected by others (classmates, neighbors, etc.) because of the parent’s incarceration. | □ | □ |  |
| 2.6. Suicidal Ideation / Self-harm | a. The child has attempted to deliberately hurt themselves. | □ | □ |  |
|  | b. The child has engaged in behavior intended to end their own life. | □ | □ |  |
|  | c. The child has expressed a wish to die or to go to sleep and not wake up. | □ | □ |  |

3. **Social Domain**

|  |  | Yes | No | Remarks |
| --- | --- | --- | --- | --- |
| 3.1. Social Functioning | a. The child gets along well with peers of the same age. | □ | □ |  |
|  | b. The child has been bullied at school. | □ | □ |  |
|  | c. The child is currently in a romantic relationship. | □ | □ |  |
|  | d. The child has participated in juvenile gang or triad activities. | □ | □ |  |
|  | e. The child is obsessed with gaming, hindering their daily routine. | □ | □ |  |
| 3.2. School Functioning | a. The child can barely keep up with schoolwork. | □ | □ |  |
|  | b. The child attends school for five consecutive days. | □ | □ |  |
|  | c. The child frequently refuses to go to school. | □ | □ |  |
|  | d. The child frequently receives complaints from teachers. | □ | □ |  |
|  | e. The child frequently gets into fights with other children or classmates. | □ | □ |  |
|  | f. The child engages in stealing behavior. | □ | □ |  |
| If the child has siblings, please complete section 3.3 below. | | | | |
| 3.3. Sibling Relationships | a. The child gets along well with siblings. | □ | □ |  |
|  | b. The child frequently has serious conflicts with their siblings. | □ | □ |  |

4. **Parenting Capacity**

|  | Yes | No | Remarks |
| --- | --- | --- | --- |
| a. I find it difficult to track the child's daily schedule. | □ | □ |  |
| b. The child is often at home without adult supervision. | □ | □ |  |
| c. The child's primary caregiver changes frequently. | □ | □ |  |

Part Two: The following questions are intended to gain a more comprehensive understanding of the child's developmental experiences and may be sensitive. If you feel uncomfortable answering at any time, please let me know immediately. Please think back to whether any of the following situations have occurred from the child’s birth up to the present (or since the child has been under your care):

5. **Abuse/Neglect**

|  |  | Yes | No | Remarks |
| --- | --- | --- | --- | --- |
| 5.1. Physical Abuse | a. A family member has physically punished the child, whether for a misbehavior or for no reason. | | | |
|  | □ Yes, including the following:  □ Spanking the child's buttocks with a hand  □ Hitting the child's buttocks with an object  □ Slapping the child's hands, arms, legs, or other body parts  □ Hitting the child's hands, arms, legs, or other body parts with a hand  □ Hitting the child's hands, arms, legs, or other body parts with an object  □ Hitting the child's head or face with an object  □ Pinching the child  □ Shaking the child  □ Other: ＿＿＿＿＿＿＿ | | □ |  |
| 5.2. Psychological Abuse | a. Family members frequently say insulting or hurtful things to the child. | □ | □ |  |
|  | b. I feel that someone in the household resents the child. | □ | □ |  |
|  | c. I have thought, "It would have been better if the child had never been born." | □ | □ |  |
|  | d. I often tell the child that he/she has inherited negative traits from the incarcerated parent. | □ | □ |  |
| 5.3. Sexual Assault | a. I believe the child has been sexually assaulted or inappropriately touched. | | | |
|  | □ Yes, including the following:  □ Someone touched the child in a sexual way, or made the child touch them.  □ Someone has made the child perform or watch sexual acts.  □ The child was sexually assaulted.  □ Someone threatened to harm or slander the child to force sexual activity.  □ Someone told the child dirty jokes.  □ The child was forced to watch pornographic videos or images.  □ Other: ＿＿＿＿＿＿＿ | | □ |  |
| 5.4. Neglect (Emotional) | a. The child rarely feels loved by the family. | □ | □ |  |
|  | b. The family has never treated the child as a real family member. | □ | □ |  |
| 5.5. Neglect (Physical) | a. The child is often unsupervised, and no one ensures their safety. | □ | □ |  |
|  | b. The child lacks sufficient food or basic hygiene care. | □ | □ |  |

6. **Matters Related to Early Juvenile Delinquency**

|  | Yes | No | Remarks |
| --- | --- | --- | --- |
| a. The child throws temper tantrums when frustrated. | □ | □ |  |
| b. The child destroys objects when frustrated. | □ | □ |  |
| c. The child becomes aggressive towards others when frustrated. | □ Yes, he/she will:  □ Attack others with hands  □ Attack using a weapon  □ Other: ＿＿＿＿＿＿ | □ |  |

__________

**End of Parent/Primary Caregiver Risk Assessment Checklist. If any questions made you feel uncomfortable, please inform the social worker or staff member. We can discuss further and provide assistance.**

# Section 8: Checklist (Child Version)

**Instructions for Completion**

This section is divided into two parts:

**Part One**: Please answer based on the situation over the past two months

This part asks about your recent life at school and at home.

**Part Two**: Please answer based on all experiences the child has had from birth up to the present

This part contains more sensitive childhood experiences (e.g., physical/psychological abuse, sexual abuse, neglect, etc.). If you feel uncomfortable while answering, please let me know, and I will handle the situation as appropriate.

__________

Part One: Please answer based on your experiences over the past two months. Please tick "Yes" or "No". If none of the situations apply, tick "No". Please provide remarks where necessary. Please note that in this section, "frequently" or “often” means three or more days out of seven.

1. **Physical Domain**

|  |  | Yes | No | Remarks |
| --- | --- | --- | --- | --- |
| 1.1. Personal Hygiene | a. I wash my hair regularly (at least every other day). | □ | □ |  |
|  | b. I brush my teeth regularly (twice daily). | □ | □ |  |
| 1.2. Sleep | a. I often want to stay up late. | □ | □ |  |
|  | b. I often have difficulty falling asleep. | □ | □ |  |
|  | c. I sleep a lot. | □ | □ |  |
|  | d. I like to take naps often. | □ | □ |  |
|  | e. I often wet the bed. | □ | □ |  |
| 1.3. Nutrition | a. I have 2-3 proper meals a day. | □ | □ |  |
|  | b. My meals include meat, vegetables, and rice. | □ | □ |  |
|  | c. I don’t like eating meals because they don’t taste good, so I don’t eat much. | □ | □ |  |

2. **Psychological Domain**

|  |  | Yes | No | Remarks |
| --- | --- | --- | --- | --- |
| 2.1. Emotions | a. I feel irritable / sad / unmotivated to do anything almost every day. | □ | □ |  |
|  | b. I worry almost every day about what will happen next. | □ Yes, worries:  ＿＿＿ | □ |  |
|  | c. I feel that I am good for nothing. | □ | □ |  |
|  | d. I feel hopeless. | □ | □ |  |
|  | e. I feel ashamed. | □ | □ |  |
| 2.2. Post-traumatic Stress Disorder (PTSD) | a. I avoid places that remind me of bad memories. | □ | □ |  |
|  | b. I frequently have nightmares or unpleasant dreams. | □ | □ |  |
|  | c. I have become very easily startled or scared suddenly (e.g., if someone approaches me unexpectedly). | □ | □ |  |
| 2.3. Attachment / Trust | a. I have become increasingly clingy to my primary caregiver. | □ | □ |  |
|  | b. I can share anything with my caregiver. | □ | □ |  |
|  | c. I try not to get too close to my caregiver. | □ | □ |  |
|  | d. I don’t want to cooperate with my caregiver. | □ | □ |  |
|  | e. I cannot trust anyone at home. | □ | □ |  |
|  | f. I have never been asked for my opinion, and no one cares what I think. | □ | □ |  |
| 2.4. Concealment and Disclosure | a. Do you know where your (incarcerated parent) is now? | | | |
|  | ☐ Yes, my parent is in ＿＿＿＿＿＿.  How did you find out? ☐ Someone told me (e.g., ＿＿＿＿＿＿＿＿＿＿) ☐ I guessed myself ☐ Other ways: ＿＿＿＿ | | □  I don’t know |  |
|  | b. If you know your parent is incarcerated, do you agree with the following statements? | | | |
|  | i. I feel people at home are unwilling to talk about him/her. | □ | □ |  |
|  | ii. I believe the reason why the parent is imprisoned. | □ | □ |  |
|  | iii. I know when he/she will be released. | □ | □ |  |
|  | iv. I have seen my parent being arrested. | □ | □ |  |
| 2.5. Contact with Incarcerated Parent | (Only for children who know their parent is incarcerated)   a. I have visited the incarcerated parent. | | | |
|  | □ Yes, visit frequency:  □ Once a week  □ Once every two weeks  □ Once a month  □ Once every three months  □ Once every six months  □ Once a year or less  Was it my own decision?  □ Yes  □ No | | □ |  |
|  | b. I keep in touch with the incarcerated parent through letters, phone calls, etc. | □ | □ |  |
| 2.6. Suicidal Ideation / Self-harm | a. I have wished I were dead, or to fall asleep and not wake up. | □ | □ |  |
|  | b. I have deliberately hurt myself. | □ | □ |  |
|  | c. I have wanted to end my own life. | □ | □ |  |

3. **Social Domain**

|  |  | Yes | No | Remarks |
| --- | --- | --- | --- | --- |
| 3.1. Social Functioning | a. I hardly get along with other kids. | □ | □ |  |
|  | b. I am often bullied by classmates. | □ | □ |  |
|  | c. I have a boyfriend or girlfriend. | □ | □ |  |
|  | d. I spend most of my time hanging out with friends on the street. | □ | □ |  |
|  | e. I spend a lot of time every day playing video games. | □ | □ |  |
| 3.2. School Functioning | a. I can barely keep up with schoolwork. | □ | □ |  |
|  | b. I often don't want to go to school. | □ | □ |  |
|  | c. I am often scolded or complained about by teachers. | □ | □ |  |
|  | d. I often get into fight with classmates. | □ | □ |  |
|  | e. I steal things. | □ | □ |  |
| If the child has siblings, please complete section 3.3 below. | | | | |
| 3.3. Sibling Relationships | a. I get along well with my siblings. | □ | □ |  |
|  | b. I often have serious conflicts with my siblings. | □ | □ |  |

4. **Parenting Capacity**

|  | Yes | No | Remarks |
| --- | --- | --- | --- |
| a. My caregiver often forgets where I am or what I'm doing. | □ | □ |  |
| b. I am often at home alone, without any adults. | □ | □ |  |
| c. I have had several different caregivers recently. | □ | □ |  |

Part Two: The following questions are intended to gain a more comprehensive understanding of your childhood experiences and may be sensitive. If you feel uncomfortable answering at any time, please tell me right away. Please answer based on your experiences as far back as you can remember:

5. **Abuse/Neglect**

|  |  | Yes | No | Remarks |
| --- | --- | --- | --- | --- |
| 5.1. Physical Abuse | a. In the past two months, I have been hit by a caregiver or family member because I did something wrong, did something unsatisfactory, or for no reason at all. | | | |
|  | □ Yes, the person has:  □ Spanked my buttocks with a hand  □ Hit my buttocks with an object  □ Hit my hands, arms, legs, or other body parts with a hand  □ Hitting the child's hands, arms, legs, or other body parts with an object  □ Pinched me  □ Shaken me  □ Other: ＿＿＿＿＿＿＿ | | □ |  |
| 5.2. Psychological Abuse | a. People at home often say insulting or hurtful things to me (e.g., "stupid," "lazy," "ugly"). | □ | □ |  |
|  | b. I have always felt that someone at home dislikes me. | □ | □ |  |
|  | c. Someone has said to me, "No one wants you." | □ | □ |  |
|  | d. I am often compared negatively to the incarcerated parent/not living at home. | □ | □ |  |
| 5.3. Sexual Assault | a. I feel/suspect that I have been sexually abused or forced to do things related to sex. | | | |
|  | □ Yes, including the following:  □ Someone touched me sexually or made me touch them sexually.  □ Someone has wanted me to do or watch things related to sex.  □ Someone has sexually assaulted me.  □ Someone threatened to harm or slander me unless I do sexual things with them.  □ Someone forced me to have sex with them.  □ Someone told me dirty jokes that I did not want to hear.  □ I have been forced to watch pornographic videos or images.  □ Other: ＿＿＿＿＿＿＿ | | □ |  |
| 5.4. Neglect (Emotional) | a. I have never felt loved. | □ | □ |  |
|  | b. I feel I don’t really count as part of the family. | □ | □ |  |
| 5.5. Neglect (Physical) | a. My caregiver provides me well with basic needs like food, clothing, and medical care. | □ | □ |  |
|  | b. When I need it, I'm not sure if anyone will come to protect me. | □ | □ |  |

6. **Matters Related to Early Juvenile Delinquency**

|  | Yes | No | Remarks |
| --- | --- | --- | --- |
| a. I lose my temper when I am frustrated. | □ | □ |  |
| b. I break things when I am frustrated. | □ | □ |  |
| c. I hit people when I am frustrated. | □ Yes, because: ＿＿＿＿＿＿＿＿＿＿＿＿. | □ |  |

7. **Perceived Stigma**

|  | Yes | No | Remarks |
| --- | --- | --- | --- |
| a. I have felt looked down upon or mocked by certain authority figures/teachers. | □ | □ |  |
| b. I have been rejected by classmates, friends, neighbors, relatives, or professionals. | □ Yes, because: ＿＿＿＿＿＿＿＿＿＿＿＿. | □ |  |

**__________**

**End of Child Version. If you felt uncomfortable while answering, or if you need to discuss anything further, please let the social worker or staff member know. We will discuss the matter with you and provide assistance.**
