# Supplementary material for: Development and pilot testing of a risk assessment checklist for children of incarcerated parents in Hong Kong
Source: Front Sociol. 2026 Sep 24;11:1936866. doi: 10.3389/fsoc.2026.1936866 (PMC13647460; doi:10.3389/fsoc.2026.1936866)
Supplement: Supplementary file 3 [file Supplementary_file_3.DOCX]

Supplementary Material

This file contains the scoring instructions for the Chinese version of the Risk Assessment Checklist, with a total of 11 pages.

# 藍巴士賽馬會結伴成長計劃 - 兒童風險評估檢核表（6–11歲）- 評分說明

1. **評分說明**

以下為兒童風險評估檢核表第二、六、七、八部分之評分說明。該部分設有「是」或「否」選項，每一項回答均對應以下三類風險等級：

- 綠色：無明顯風險
- 黃色：存在部分風險，建議持續觀察或給予所需支援
- 紅色：風險較高，需進一步深入評估或即時介入

請根據填答結果，依上述分類進行標記與後續處理。

2. **關注程度分類**

| 風險等級類別 | 分流準則 | 跟進介入 |
| --- | --- | --- |
| 紅色 （高度關注）  從檢核表中填答，已顯示了服務需要 | 第二部份：孩子個人資料   1. 如在以下題目選擇了「是」, 即時跟進   題目包括：11  第六部份：社工家訪觀察   1. 如在以下題目選擇了「是」, 即時跟進   題目包括：10  第七部份：檢核表（家長/主要照顧者版本）   1. 如在以下題目選擇了「是」, 即時跟進   題目包括：2.2 a-c, 2.6 a-c, 3.1b, 5.1 a, 5.2 a-d, 5.3 a, 5.4 a-b, 5.5 a-b, 6 a-c  第八部份：檢核表（兒童版本）   1. 如在以下題目選擇了「是」, 即時跟進   題目包括：2.2 a-c, 2.6 a-c, 3.1b, 5.1 a, 5.2 a-d, 5.3 a, 5.4 a-b, 5.5 a-b, 6 a-c | 即時跟進： 進行獨立評估，按需要以個案跟進。 |
| 黃色 （中度關注）  從檢核表中填答，可能顯示了有潛在危機。 | 第二部份：孩子個人資料   1. 如在以下題目選擇了「是」, 即時跟進   題目包括：10  第六部份：社工家訪觀察   1. 如在以下題目選擇了「是」, 持續觀察   題目包括：5, 12   2. 如在以下題目選擇了「否」, 持續觀察   題目包括：1-4, 6-10  第七部份：檢核表（家長/主要照顧者版本）   1. 如在以下題目選擇了「是」, 持續觀察   題目包括：1.2 a-e, 1.3 b-d, 2.1 a-b, 2.3 a, 2.3 c, 2.4 a-c, 2.5 d, 3.1 c-e, 3.2 a, 3.2 c-g, 3.3 b, 4 a-c   2. 如在以下題目選擇了「否」, 持續觀察   題目包括：1.1 a-b, 1.3 a, 2.3 b, 2.4 d, 2.5 a-c, 3.1 a, 3.1 c, 3.2 b, 3.3 a  第八部份：檢核表（兒童版本）   1. 如在以下題目選擇了「是」, 持續觀察   題目包括：1.2 a-d, 1.3 c, 2.1 a-e, 2.3 a, 2.3 c-f, 2.4 b.1, 2.4 b.4, 3.1 a, 3.1 c-e, 3.2 a-e, 3.3 b, 4 a-c, 7 a-b   2. 如在以下題目選擇了「否」, 持續觀察   題目包括：1.1 a-b, 1.3 a-b, 2.3 b, 2.4 a, 2.4 b.2-3, 2.5 a-b, 3.1 c, 3.3a | 持續觀察：  需要進一步提問詳情或搜集資料，以評估風險的程度及提供所需支援 |
| 綠色 （低度關注）  從檢核表中填答，沒有潛在危機和即時需要。 | 第二、六、七、八部分填答結果不含危機因素。 | 跟進方案：  暫時毋須跟進 |

# 藍巴士賽馬會結伴成長計劃 - 兒童風險評估檢核表（6–11歲）

# 第二部份：孩子個人資料

10. 孩子是否有確診的特殊教育需要/心理或精神健康問題 ?

(例如：智力和發展障礙、自閉症譜系障礙、注意力不足或過度活躍、讀寫障礙)

□ 否

□ 是

特殊教育需要 / 心理或精神健康問題紀錄:_________________

確診單位/機構: _________________

確診年份: _________________

支援服務: _______________________________ □ 不適用

藥物治療: _________________ □ 不適用 (是否定期服用:是□，否□ )

特殊學校就讀情況：是□，否□

11. 孩子有沒有因懷疑被傷害/虐待而曾召開「保護懷疑受虐待兒童多專業個案會議(MDCC)」?

□ 有，請簡述發生了什麼事情：__________________________

□ 否

□ 不知道

# 第六部份：社工家訪觀察

|  | 是 | 否 | 備註 |
| --- | --- | --- | --- |
| 1. 住處看起來安全。 | □ | □ |  |
| 2. 住處大致乾淨。 | □ | □ |  |
| 3. 住處光線充足。 | □ | □ |  |
| 4. 住處有基本設施，供孩子睡覺、玩耍、學習和休息。 | □ | □ |  |
| 5. 孩子與其他人（父母、異性等）共用一張床。 | □ | □ | 若是, 請說明與誰以及年齡差距：＿＿＿＿＿＿＿＿＿＿＿＿＿＿＿＿＿＿＿ |
| 6. 住處為孩子提供個人空間（如床位、書櫃、衣櫃）。 | □ | □ |  |
| 7. 住處有烹飪設備，並確實用於烹煮孩子與家人所需的膳食。 | □ | □ |  |
| 8. 從街道到住處的通道光線充足且暢通。 | □ | □ |  |
| 9. 社區環境有一些兒童遊樂或休閒設施。 | □ | □ |  |
| 10. 孩子穿著乾淨且合身的衣物。 | □ | □ |  |
| 11. 孩子有明顯的傷痕或傷口。 | □ | □ |  |
| 12. 住處經常有非親屬或孩子不熟悉的成年人出入。 | □ | □ |  |

# 第七部份：檢核表（家長/主要照顧者版本）

1. **生理範疇**

|  |  | 是 | 否 | 備註 |
| --- | --- | --- | --- | --- |
| 1.1. 個人衛生 | a. 孩子定期洗頭（至少隔日）。 | □ | □ |  |
|  | b. 孩子定期刷牙（每天 2 次）。 | □ | □ |  |
| 1.2. 睡眠 | a. 孩子經常拒絕上床睡覺。 | □ | □ |  |
|  | b. 孩子經常難以入睡。 | □ | □ |  |
|  | c. 我的孩子經常睡得很久。 | □ | □ |  |
|  | d. 我的孩子常常小睡。 | □ | □ |  |
|  | e. 孩子在睡覺時經常尿床。 | □ | □ |  |
| 1.3. 營養 | a. 孩子都有定時享用正餐。 | □ | □ |  |
|  | b. 我的孩子吃得很少。 | □ | □ |  |
|  | c. 孩子常以零食取代正餐。 | □ | □ |  |
|  | d. 我的孩子有挑食的習慣。 | □ | □ |  |

2. **心理範疇**

|  |  | 是 | 否 | 備註 |
| --- | --- | --- | --- | --- |
| 2.1. 情緒 | a. 孩子總是煩躁/悲傷/缺乏動力和興趣。 | □ | □ |  |
|  | b. 孩子總是顯得擔憂/缺乏安全感。 | □ | □ |  |
| 2.2. 創傷後壓力症(PTSD) | a. 孩子會迴避任何引起創傷回憶的人、地點或活動。 | □ | □ |  |
|  | b. 孩子經常發惡夢或有不愉快的夢境。 | □ | □ |  |
|  | c. 孩子變得容易受到驚嚇（例如有人從背後靠近時會嚇一跳）。 | □ | □ |  |
| 2.3. 依附關係 | a. 孩子變得越來越黏我。 | □ | □ |  |
|  | b. 孩子願意與我坦誠分享任何事。 | □ | □ |  |
|  | c. 孩子刻意避免與我親近。 | □ | □ |  |
| 2.4. 遮瞞與坦白 | a. 我一直避免提及在囚家長的缺席。 | □ | □ |  |
|  | b. 我會編造理由（如工作、旅行）來解釋在囚家長的離開。 | □ | □ |  |
|  | c. 孩子曾目睹該家長被逮捕。 | □ | □ |  |
|  | d. 我有向孩子解釋過，他/她的家長正在服刑。 | □ | □ |  |
| 2.5. 與在囚家長的聯繫 | a. 孩子知道家長何時會釋放。 | □ | □ |  |
|  | b. 我讓孩子自己決定何時探望在囚家長。 | □ | □ |  |
|  | c. 在囚家長與孩子定期以不同方式（如信件、探視、電話）保持聯繫。 | □ | □ |  |
|  | d. 孩子曾表示因家長被囚而遭到他人排斥（同學、鄰居等）。 | □ | □ |  |
| 2.6. 自殺念頭/自殘行為 | a. 孩子曾嘗試故意傷害自己。 | □ | □ |  |
|  | b. 孩子曾做過想結束自己生命的行為。 | □ | □ |  |
|  | c. 孩子曾表示希望自己死去或一睡不醒。 | □ | □ |  |

3. **社交範疇**

|  |  | 是 | 否 | 備註 |
| --- | --- | --- | --- | --- |
| 3.1. 社交功能 | a. 孩子和同齡朋友相處良好。 | □ | □ |  |
|  | b. 孩子曾在學校受到欺凌。 | □ | □ |  |
|  | c. 孩子目前有戀愛關係。 | □ | □ |  |
|  | d. 孩子曾參與童黨或幫派活動。 | □ | □ |  |
|  | e. 孩子沉迷於遊戲，妨礙日常作息。 | □ | □ |  |
| 3.2. 學校生活功能 | a. 孩子幾乎跟不上課業進度。 | □ | □ |  |
|  | b. 孩子能連續五天都去上學。 | □ | □ |  |
|  | c. 孩子經常不願意上學。 | □ | □ |  |
|  | d. 孩子經常被老師投訴。 | □ | □ |  |
|  | e. 孩子常與其他兒童或同學打架。 | □ | □ |  |
|  | f. 孩子有偷竊的行為。 | □ | □ |  |
| 3.3. 兄弟姐妹關係 | a. 孩子與兄弟姐妹相處融洽。 | □ | □ |  |
|  | b. 孩子與兄弟姐妹常有嚴重衝突。 | □ | □ |  |

4. **家長職能**

|  | 是 | 否 | 備註 |
| --- | --- | --- | --- |
| a. 我覺得很難追蹤孩子每天的行程。 | □ | □ |  |
| b. 孩子經常在家卻沒有成人照顧。 | □ | □ |  |
| c. 孩子主要的照顧者經常更換。 | □ | □ |  |

5. **與虐待/疏忽照顧有關**

|  |  | 是 | 否 | 備註 |
| --- | --- | --- | --- | --- |
| 5.1. 身體虐待 | a. 家人曾因孩子犯錯/無故而對孩子使用體罰。 | | | |
|  | □ 是 ，包含下列情況：  □ 用手掌打孩子臀部  □ 用物件打孩子臀部  □ 拍打孩子手、臂、腿或其他部位  □ 用物件打孩子手、臂、腿或其他部位  □ 用物件打孩子頭部或臉部  □ 捏孩子  □ 搖晃孩子  □ 其他：＿＿＿＿＿＿＿ | | □ |  |
| 5.2. 心理虐待 | a. 家人經常對孩子說帶有侮辱或傷害性的話。 | □ | □ |  |
|  | b. 我感覺家中有人討厭孩子。 | □ | □ |  |
|  | c. 我曾想過「如果孩子沒出生就好了」。 | □ | □ |  |
|  | d. 我常跟孩子說，他/她遺傳了被囚家長的負面特質。 | □ | □ |  |
| 5.3. 性侵犯 | a. 我相信孩子曾遭性侵犯或不當性接觸。 | | | |
|  | □ 是, 包含下列情況：  □ 有人曾以性方式觸碰孩子，或讓孩子觸碰他們。  □ 有人曾要求孩子做或觀看色情行為。  □ 孩子曾實際遭到性侵犯。  □ 有人以傷害或誣衊相威脅，逼迫孩子進行性行為。  □ 有人曾對孩子講黃色笑話。  □ 孩子曾被迫觀看色情影片或圖片。  □ 其他：＿＿＿＿＿＿＿ | | □ |  |
| 5.4. 疏忽照顧(情感) | a. 孩子很少感受到家人的關愛。 | □ | □ |  |
|  | b. 家人從未把孩子當作真正的家庭成員。 | □ | □ |  |
| 5.5. 疏忽照顧(身體) | a. 孩子經常沒有人照顧，也無人確保其安全。 | □ | □ |  |
|  | b. 孩子缺乏足夠的食物或基本生活/衛生照料。 | □ | □ |  |

6. **早期青少年犯罪**

|  | 是 | 否 | 備註 |
| --- | --- | --- | --- |
| a. 孩子沮喪時會亂發脾氣。 | □ | □ |  |
| b. 孩子沮喪時會破壞物品。 | □ | □ |  |
| c. 孩子沮喪時會攻擊他人。 | □ 是，他/她會：  □ 用手攻擊他人  □ 使用武器攻擊  □ 其他：＿＿＿＿＿ | □ |  |

# 第八部份：檢核表（兒童版本）

1. **生理範疇**

|  |  | 是 | 否 | 備註 |
| --- | --- | --- | --- | --- |
| 1.1. 個人衛生 | a. 我會定期洗頭（至少隔日）。 | □ | □ |  |
|  | b. 我會定期刷牙（每天2次）。 | □ | □ |  |
| 1.2. 睡眠 | a. 我常常想要晚一點才睡。 | □ | □ |  |
|  | b. 我常常難以入睡。 | □ | □ |  |
|  | c. 我睡得很多。 | □ | □ |  |
|  | d. 我喜歡常常小睡。 | □ | □ |  |
|  | e. 我經常尿床。 | □ | □ |  |
| 1.3. 營養 | a. 我每天有2-3次正餐。 | □ | □ |  |
|  | b. 我的正餐有肉、蔬菜和米飯。 | □ | □ |  |
|  | c. 我不愛吃飯，因為不好吃，所以吃得不多。 | □ | □ |  |

2. **心理範疇**

|  |  | 是 | 否 | 備註 |
| --- | --- | --- | --- | --- |
| 2.1. 情緒 | a. 我幾乎每天都感到煩躁/悲傷/沒心情做事。 | □ | □ |  |
|  | b. 我幾乎每天都在擔心之後會發生什麼。 | □是,擔心：  ＿＿＿＿ | □ |  |
|  | c. 我覺得自己一無是處。 | □ | □ |  |
|  | d. 我感到絕望。 | □ | □ |  |
|  | e. 我感到羞恥。 | □ | □ |  |
| 2.2. 創傷後壓力症(PTSD) | a. 我會避開讓我想起不好回憶的地方。 | □ | □ |  |
|  | b. 我經常發惡夢或有不愉快的夢境。 | □ | □ |  |
|  | c. 我變得非常容易受到驚嚇或突然被嚇到（例如有人突然靠近我）。 | □ | □ |  |
| 2.3. 依附關係/信任 | a. 我變得越來越黏主要照顧者。 | □ | □ |  |
|  | b. 我可以跟照顧者分享任何事。 | □ | □ |  |
|  | c. 我盡量不和照顧者走得太近。 | □ | □ |  |
|  | d. 我不想跟照顧者合作。 | □ | □ |  |
|  | e. 我無法信任家裡的任何人。 | □ | □ |  |
|  | f. 我從來沒有被問過意見，也沒有人關心我在想什麼。 | □ | □ |  |
| 2.4. 遮瞞與坦白 | a. 你知道你的（在囚家長）現在在哪裡嗎？ | | | |
|  | ☐ 知道，我的家長在 ＿＿＿＿＿。  你是怎樣知道的？ ☐ 有人告訴我（例如：＿＿＿＿＿＿＿＿＿＿） ☐ 自己猜的 ☐ 其他方式：＿＿＿＿＿＿ | | ☐ 不知道 |  |
|  | b. 如果你知道家長**被囚禁**，以下說法你是否同意？ | | | |
|  | i. 如果我知道家長被囚：我感覺家裡的人都不願意談論他/她。 | □ | □ |  |
|  | ii. 如果我知道家長被囚：我相信家長被監禁的理由。 | □ | □ |  |
|  | iii. 如果我知道家長被囚：我知道他 / 她什麼時候會出來。 | □ | □ |  |
|  | iv. 我曾經親眼看見家長被逮捕。 | □ | □ |  |
| 2.5. 與在囚家長的聯繫 | a. 我有去探望被囚禁的家長。 | | | |
|  | □ 有，探訪頻率：  □ 每星期一次  □ 每兩星期一次  □ 每月一次  □ 每三個月一次  □ 每半年一次  □ 每年一次或更少  是我自己決定的嗎？  □ 是  □ 不是 | | □ 沒有 |  |
|  | b. 我透過信件或電話等方式和被囚禁的家長保持聯繫。 | □ | □ |  |
| 2.6. 自殺念頭/自殘行為 | a. 我曾經希望自己死掉，或睡了就不要再醒來。 | □ | □ |  |
|  | b. 我曾故意傷害自己。 | □ | □ |  |
|  | c. 我曾想結束自己的生命。 | □ | □ |  |

3. **社交範疇**

|  |  | 是 | 否 | 備註 |
| --- | --- | --- | --- | --- |
| 3.1. 社交功能 | a. 我幾乎和其他孩子相處不來。 | □ | □ |  |
|  | b. 我經常被同學欺負。 | □ | □ |  |
|  | c. 我有男朋友或女朋友。 | □ | □ |  |
|  | d. 我大多時間都在街上跟朋友混在一起。 | □ | □ |  |
|  | e. 我每天花很多時間打電動。 | □ | □ |  |
| 3.2. 學校生活功能 | a. 我幾乎跟不上學校的課業。 | □ | □ |  |
|  | b. 我常常不想上學。 | □ | □ |  |
|  | c. 我常常被老師罵或投訴。 | □ | □ |  |
|  | d. 我常常跟同學打架。 | □ | □ |  |
|  | e. 我有偷竊的行為。 | □ | □ |  |
| 3.3. 兄弟姐妹關係 | a. 我跟兄弟姐妹相處融洽。 | □ | □ |  |
|  | b. 我跟兄弟姐妹常有嚴重衝突。 | □ | □ |  |

4. **家長職能**

|  | 是 | 否 | 備註 |
| --- | --- | --- | --- |
| a. 我的照顧者常常忘記我在哪裡、在做什麼。 | □ | □ |  |
| b. 我時常一個人在家，沒有任何大人。 | □ | □ |  |
| c. 最近我換了好幾位照顧者。 | □ | □ |  |

5. **與虐待/疏忽照顧有關**

|  |  | 是 | 否 | 備註 |
| --- | --- | --- | --- | --- |
| 5.1. 身體虐待 | a. 過去兩個月，我因為做錯事而被照顧者或家人打。 | | | |
|  | □ 是，對方曾經：  □ 用手掌打我屁股  □ 用物件打我屁股  □ 用手掌打我手、手臂、腿或身體其他部位  □ 用物件打我手、手臂、腿或身體其他部位  □ 用物件打我頭部或臉部  □ 捏我  □ 搖晃我  □ 其他：＿＿＿＿＿＿＿ | | □ |  |
| 5.2. 心理虐待 | a. 家裡的人經常對我說侮辱或傷害性的話（例如「笨」「懶」「醜」）。 | □ | □ |  |
|  | b. 我一直感覺家裡有人討厭我。 | □ | □ |  |
|  | c. 有人對我說過「沒人想要你」。 | □ | □ |  |
|  | d. 我經常被拿來和入獄的家長做負面比較。 | □ | □ |  |
| 5.3. 性侵犯 | a. 我覺得自己曾遭到性侵犯或被迫做與性有關的事 | | | |
|  | □ 是，包含下列情況：  □ 有人曾以性方式碰我，或想讓我碰他們。  □ 有人想要我做或觀看與性有關的事情。  □ 有人對我進行了性侵犯。  □ 有人威脅要傷害或誣衊我，除非我跟他 / 她做某些性行為。  □ 有成人強迫我和他 / 她進行性行為。  □ 有人跟我說過黃色笑話，我並不想聽。  □ 我曾被迫觀看色情影片或圖片。  □ 其他：＿＿＿＿＿＿＿ | | □ |  |
| 5.4. 疏忽照顧(情感) | a. 我從來沒有感受到被愛。 | □ | □ |  |
|  | b. 我覺得自己不算是家裡的一份子。 | □ | □ |  |
| 5.5. 疏忽照顧(身體) | a. 我的照顧者有好好給我食物、衣服、醫療等基本需求。 | □ | □ |  |
|  | b. 當我需要時，我不確定有沒有人會來保護我。 | □ | □ |  |

6. **早期青少年犯罪**

|  | 是 | 否 | 備註 |
| --- | --- | --- | --- |
| a. 我在沮喪時會發脾氣。 | □ | □ |  |
| b. 我在沮喪時會破壞東西。 | □ | □ |  |
| c. 我在沮喪時會打人。 | □是, 因為:  ___________________ | □ |  |

7. **污名感知**

|  | 是 | 否 | 備註 |
| --- | --- | --- | --- |
| a. 我曾覺得自己被某些權威 / 師長看輕或取笑。 | □ | □ |  |
| b. 我曾被同學、朋友、鄰居、親戚或專業人士排斥。 | □是, 因為:  ___________________ | □ |  |
