# Supplementary material for: Development and pilot testing of a risk assessment checklist for children of incarcerated parents in Hong Kong
Source: Front Sociol. 2026 Sep 24;11:1936866. doi: 10.3389/fsoc.2026.1936866 (PMC13647460; doi:10.3389/fsoc.2026.1936866)
Supplement: Supplementary file 4 [file Supplementary_file_4.DOCX]

Supplementary Material

This file contains the scoring instructions for the Chinese version of the Risk Assessment Checklist, with a total of 14 pages.

**Blue Bus Jockey Club Together We Grow Project – Child Risk Assessment Checklist (Aged 6-11) – Scoring Instructions**

1. **Scoring Instructions**

The following are scoring instructions for Sections 2, 6, 7, and 8 of the Child Risk Assessment Checklist. These sections include "Yes" or "No" response options, with each answer corresponding to one of the three risk levels below:

- Green: No significant risk identified
- Yellow: Some risk identified, continued observation or provision of necessary support is recommended
- Red: Higher risk, further in-depth assessment or immediate intervention is required

Based on the responses, please mark the corresponding risk level and proceed with follow-up actions accordingly.

2. **Risk Level Classification**

| Risk Level | Criteria | Follow-up Actions |
| --- | --- | --- |
| Red (High concern) | Section 2: Child's Personal Information   1. Immediate follow-up if “Yes” is selected for:   Item 11  Section 6: Social Worker Home Visit Observations   1. Immediate follow-up if “Yes” is selected for:   Item 10  Section 7: Checklist (Parent/Primary Caregiver Version)   1. Immediate follow-up if “Yes” is selected for:   Items 2.2 a-c, 2.6 a-c, 3.1b, 5.1 a, 5.2 a-d, 5.3 a, 5.4 a-b, 5.5 a-b, 6 a-c  Section 8: Checklist (Child Version)   1. Immediate follow-up if “Yes” is selected for:   Items 2.2 a-c, 2.6 a-c, 3.1b, 5.1 a, 5.2 a-d, 5.3 a, 5.4 a-b, 5.5 a-b, 6 a-c | Immediate follow-up: Conduct an independent assessment and provide case follow-up as needed. |
| Yellow (Moderate concern) | Section 2: Child's Personal Information   1. Immediate follow-up if “Yes” is selected for:   Item 10  Section 6: Social Worker Home Visit Observations   1. Continued observation if “Yes” is selected for:   Items 5, 12   2. Continued observation if “No” is selected for:   Items 1-4, 6-10  Section 7: Checklist (Parent/Primary Caregiver Version)   1. Continued observation if “Yes” is selected for:   Items 1.2 a-e, 1.3 b-d, 2.1 a-b, 2.3 a, 2.3 c, 2.4 a-c, 2.5 d, 3.1 c-e, 3.2 a, 3.2 c-g, 3.3 b, 4 a-c   2. Continued observation if “No” is selected for:   Items 1.1 a-b, 1.3 a, 2.3 b, 2.4 d, 2.5 a-c, 3.1 a, 3.1 c, 3.2 b, 3.3 a  Section 8: Checklist (Child version)   1. Continued observation if “Yes” is selected for:   Items 1.2 a-d, 1.3 c, 2.1 a-e, 2.3 a, 2.3 c-f, 2.4 b.1, 2.4 b.4, 3.1 a, 3.1 c-e, 3.2 a-e, 3.3 b, 4 a-c, 7 a-b   2. Continued observation if “No” is selected for:   Items 1.1 a-b, 1.3 a-b, 2.3 b, 2.4 a, 2.4 b.2-3, 2.5 a-b, 3.1 c, 3.3a | Ongoing observation:  Further questioning or information gathering is needed to assess the level of risk and provide the necessary support. |
| Green (Low concern) | Responses in Sections 2, 6, 7, and 8 contain no risk factors | Follow-up plan: No follow-up required at this time |

# Blue Bus Jockey Club Together We Grow Project – Child Risk Assessment Checklist (Aged 6-11)

# Section 2: Child’s Personal Details

10. Does the child have any diagnosed Special Educational Needs (SEN) or mental health conditions?
    (e.g., intellectual and developmental disabilities, Autism Spectrum Disorder, Attention Deficit Hyperactivity Disorder, dyslexia)

□ No

□ Yes

SEN / Mental Health Condition Record: _______________

Diagnosing Institution / Organization: _________________

Year of Diagnosis: _________________

Support Services: _______________________________ □ Not Applicable

Medication: ____________ □ Not applicable (If yes, is it taken regularly? □ Yes □ No)

Attends a special needs school: □ Yes □ No

11. Has a Multi-disciplinary Case Conference (MDCC) on Suspected Child Abuse ever been convened for the child?

□ Yes, please briefly describe what happened: __________________________

□ No

□ Don’t know

# Section 6: Social Worker Home Visit Observations

|  | Yes | No | Remarks |
| --- | --- | --- | --- |
| 1. The residence appears safe. | □ | □ |  |
| 2. The residence is generally clean. | □ | □ |  |
| 3. The residence has adequate lighting. | □ | □ |  |
| 4. The residence has basic facilities for the child to sleep, play, study, and rest. | □ | □ |  |
| 5. The child shares a bed with others (parents, opposite sex, etc.). | □ | □ | If yes, please specify with whom and the age difference：＿＿＿＿＿＿＿＿＿＿ |
| 6. The residence provides personal space for the child (e.g., bed, bookshelf, wardrobe). | □ | □ |  |
| 7. The residence has cooking facilities, which are used to prepare meals for the child and family. | □ | □ |  |
| 8. The access path from the street to the residence is well-lit and unobstructed. | □ | □ |  |
| 9. The community environment has some children's playground or leisure facilities. | □ | □ |  |
| 10. The child is wearing clean and well-fitting clothes. | □ | □ |  |
| 11. The child has visible bruises or wounds. | □ | □ |  |
| 12. Non-relatives or adults unfamiliar to the child frequently visit the residence. | □ | □ |  |

# Section 7: Checklist (Parent/Primary Caregiver Version)

1. **Physical Domain**

|  |  | Yes | No | Remarks |
| --- | --- | --- | --- | --- |
| 1.1. Personal Hygiene | a. The child washes their hair regularly (at least every other day). | □ | □ |  |
|  | b. The child brushes their teeth regularly (twice daily). | □ | □ |  |
| 1.2. Sleep | a. The child frequently refuses to go to bed. | □ | □ |  |
|  | b. The child frequently has difficulty falling asleep. | □ | □ |  |
|  | c. The child frequently sleeps for long periods. | □ | □ |  |
|  | d. The child frequently naps. | □ | □ |  |
|  | e. The child frequently wets the bed during sleep. | □ | □ |  |
| 1.3. Nutrition | a. The child has regular meals on time. | □ | □ |  |
|  | b. The child eats very little. | □ | □ |  |
|  | c. The child often replaces meals with snacks. | □ | □ |  |
|  | d. The child is a picky eater. | □ | □ |  |

2. **Psychological Domain**

|  |  | Yes | No | Remarks |
| --- | --- | --- | --- | --- |
| 2.1. Emotions | a. The child is always irritable / sad / lacks motivation and interest. | □ | □ |  |
|  | b. The child always appears worried. | □ | □ |  |
| 2.2. Post-traumatic Stress Disorder (PTSD) | a. The child avoids any people, places, or activities that trigger traumatic memories. | □ | □ |  |
|  | b. The child frequently has nightmares or unpleasant dreams. | □ | □ |  |
|  | c. The child has become easily startled (e.g., jumps when someone approaches from behind). | □ | □ |  |
| 2.3. Attachment | a. The child has become increasingly clingy towards me. | □ | □ |  |
|  | b. The child is willing to share anything with me openly and honestly. | □ | □ |  |
|  | c. The child deliberately avoids being close to me. | □ | □ |  |
| 2.4. Concealment and Disclosure | a. I have been avoiding mentioning the incarcerated parent's absence. | □ | □ |  |
|  | b. I make up reasons (e.g., work, travel) to explain the incarcerated parent's absence. | □ | □ |  |
|  | c. The child has witnessed the parent’s arrest. | □ | □ |  |
|  | d. I have explained to the child that his/her parent is serving a prison sentence. | □ | □ |  |
| 2.5. Contact with Incarcerated Parent | a. The child knows when the parent will be released. | □ | □ |  |
|  | b. I let the child decide when to visit the incarcerated parent. | □ | □ |  |
|  | c. The incarcerated parent and the child maintain regular contact through various means (e.g., letters, visits, phone calls). | □ | □ |  |
|  | d. The child has reported being rejected by others (classmates, neighbors, etc.) because of the parent’s incarceration | □ | □ |  |
| 2.6. Suicidal Ideation / Self-harm | a. The child has attempted to deliberately hurt themselves. | □ | □ |  |
|  | b. The child has engaged in behavior intended to end their own life. | □ | □ |  |
|  | c. The child has expressed a wish to die or to go to sleep and not wake up. | □ | □ |  |

3. **Social Domain**

|  |  | Yes | No | Remarks |
| --- | --- | --- | --- | --- |
| 3.1. Social Functioning | a. The child gets along well with peers of the same age. | □ | □ |  |
|  | b. The child has been bullied at school. | □ | □ |  |
|  | c. The child is currently in a romantic relationship. | □ | □ |  |
|  | d. The child has participated in juvenile gang or triad activities. | □ | □ |  |
|  | e. The child is obsessed with gaming, hindering their daily routine. | □ | □ |  |
| 3.2. School Functioning | a. The child can barely keep up with schoolwork. | □ | □ |  |
|  | b. The child attends school for five consecutive days. | □ | □ |  |
|  | c. The child frequently refuses to go to school. | □ | □ |  |
|  | d. The child frequently receives complaints from teachers. | □ | □ |  |
|  | e. The child frequently gets into fights with other children or classmates. | □ | □ |  |
|  | f. The child engages in stealing behavior. | □ | □ |  |
|  | g. The child can barely keep up with schoolwork. | □ | □ |  |
| 3.3. Sibling Relationships | a. The child gets along well with siblings. | □ | □ |  |
|  | b. The child frequently has serious conflicts with their siblings. | □ | □ |  |

4. **Parenting Capacity**

|  | Yes | No | Remarks |
| --- | --- | --- | --- |
| a. I find it difficult to track the child's daily schedule. | □ | □ |  |
| b. The child is often at home without adult supervision. | □ | □ |  |
| c. The child's primary caregiver changes frequently. | □ | □ |  |

5. **Abuse/Neglect**

|  |  | Yes | No | Remarks |
| --- | --- | --- | --- | --- |
| 5.1. Physical Abuse | a. A family member has physically punished the child, whether for a misbehavior or for no reason. | | | |
|  | □ □ Yes, including the following:  □ Spanking the child's buttocks with a hand  □ Hitting the child's buttocks with an object  □ Slapping the child's hands, arms, legs, or other body parts  □ Hitting the child's hands, arms, legs, or other body parts with a hand  □ Hitting the child's hands, arms, legs, or other body parts with an object  □ Hitting the child's head or face with an object  □ Pinching the child  □ Shaking the child  □ Other: ＿＿＿＿＿＿＿ | | □ |  |
| 5.2. Psychological Abuse | a. Family members frequently say insulting or hurtful things to the child. | □ | □ |  |
|  | b. I feel that someone in the household resents the child. | □ | □ |  |
|  | c. I have thought, "It would have been better if the child had never been born." | □ | □ |  |
|  | d. I often tell the child that he/she has inherited negative traits from the incarcerated parent. | □ | □ |  |
| 5.3. Sexual Assault | a. I believe the child has been sexually assaulted or inappropriately touched. | | | |
|  | □ Yes, including the following:  □ Someone touched the child in a sexual way, or made the child touch them.  □ Someone has made the child perform or watch sexual acts.  □ The child was sexually assaulted.  □ Someone threatened to harm or slander the child to force sexual activity.  □ Someone told the child dirty jokes.  □ The child was forced to watch pornographic videos or images.  □ Other: ＿＿＿＿＿＿＿ | | □ |  |
| 5.4. Neglect (Emotional) | a. The child rarely feels loved by the family. | □ | □ |  |
|  | b. The family has never treated the child as a real family member. | □ | □ |  |
| 5.5. Neglect (Physical) | a. The child is often unsupervised, and no one ensures their safety. | □ | □ |  |
|  | b. The child lacks sufficient food or basic hygiene care. | □ | □ |  |

6. **Matters Related to Early Juvenile Delinquency**

|  | Yes | No | Remarks |
| --- | --- | --- | --- |
| a. The child throws temper tantrums when frustrated. | □ | □ |  |
| b. The child destroys objects when frustrated. | □ | □ |  |
| c. The child becomes aggressive towards others when frustrated. | □ Yes, he/she will:  □ Attack others with hands  □ Attack using a weapon  □ Other: ＿＿＿＿＿ | □ |  |

# Section 8: Checklist (Child Version)

1. **Physical Domain**

|  |  | Yes | No | Remarks |
| --- | --- | --- | --- | --- |
| 1.1. Personal Hygiene | a. I wash my hair regularly (at least every other day). | □ | □ |  |
|  | b. I brush my teeth regularly (twice daily). | □ | □ |  |
| 1.2. Sleep | a. I often want to stay up late. | □ | □ |  |
|  | b. I often have difficulty falling asleep. | □ | □ |  |
|  | c. I sleep a lot. | □ | □ |  |
|  | d. I like to take naps often. | □ | □ |  |
|  | e. I often wet the bed. | □ | □ |  |
| 1.3. Nutrition | a. I have 2-3 proper meals a day. | □ | □ |  |
|  | b. My meals include meat, vegetables, and rice. | □ | □ |  |
|  | c. I don’t like eating meals because they don’t taste good, so I don’t eat much. | □ | □ |  |

2. **Psychological Domain**

|  |  | Yes | No | Remarks | |
| --- | --- | --- | --- | --- | --- |
| 2.1. Emotions | a. I feel irritable / sad / unmotivated to do anything almost every day. | □ | □ | |  |
|  | b. I worry almost every day about what will happen next. | □ Yes, worries：  ＿＿＿＿ | □ | |  |
|  | c. I feel that I am good for nothing. |  |  | |  |
|  | d. I feel hopeless. | □ | □ | |  |
|  | e. I feel ashamed. | □ | □ | |  |
| 2.2. Post-traumatic Stress Disorder (PTSD) | a. I avoid places that remind me of bad memories. | □ | □ | |  |
|  | b. I frequently have nightmares or unpleasant dreams. | □ | □ | |  |
|  | c. I have become very easily startled or scared suddenly (e.g., if someone approaches me unexpectedly). | □ | □ | |  |
| 2.3. Attachment / Trust | a. I have become increasingly clingy to my primary caregiver. | □ | □ | |  |
|  | b. I can share anything with my caregiver. | □ | □ | |  |
|  | c. I try not to get too close to my caregiver. | □ | □ | |  |
|  | d. I don’t want to cooperate with my caregiver. | □ | □ | |  |
|  | e. I cannot trust anyone at home. | □ | □ | |  |
|  | f. I have never been asked for my opinion, and no one cares what I think. | □ | □ | |  |
| 2.4. Concealment and Disclosure | a. Do you know where your (incarcerated parent) is now? | | | | |
|  | ☐ ☐ Yes, my parent is in ＿＿＿＿＿＿.  How did you find out? ☐ Someone told me (e.g., ＿＿＿＿＿＿＿＿＿＿) ☐ I guessed myself ☐ Other ways: ＿＿＿＿ | | ☐  I don’t know | |  |
|  | b. If you know your parent is incarcerated, do you agree with the following statements? | | | | |
|  | i. I feel people at home are unwilling to talk about him/her. | □ | □ | |  |
|  | ii. I believe the reason why the parent is imprisoned. | □ | □ | |  |
|  | iii. I know when he/she will be released. | □ | □ | |  |
|  | iv. I have seen my parent being arrested. | □ | □ | |  |
| 2.5. Contact with Incarcerated Parent | a. I have visited the incarcerated parent. | | | | |
|  | □ Yes, visit frequency:  □ Once a week  □ Once every two weeks  □ Once a month  □ Once every three months  □ Once every six months  □ Once a year or less  Was it my own decision?  □ Yes  □ No | | □ | |  |
|  | b. I keep in touch with the incarcerated parent through letters, phone calls, etc. | □ | □ | |  |
| 2.6. Suicidal Ideation / Self-harm | a. I have wished I were dead, or to fall asleep and not wake up. | □ | □ | |  |
|  | b. I have deliberately hurt myself. | □ | □ | |  |
|  | c. I have wanted to end my own life. | □ | □ | |  |

3. **Social Domain**

|  |  | Yes | No | Remarks |
| --- | --- | --- | --- | --- |
| 3.1. Social Functioning | a. I hardly get along with other kids. | □ | □ |  |
|  | b. I am often bullied by classmates. | □ | □ |  |
|  | c. I have a boyfriend or girlfriend. | □ | □ |  |
|  | d. I spend most of my time hanging out with friends on the street. | □ | □ |  |
|  | e. I spend a lot of time every day playing video games. | □ | □ |  |
| 3.2. School Functioning | a. I can barely keep up with schoolwork. | □ | □ |  |
|  | b. I often don't want to go to school. | □ | □ |  |
|  | c. I am often scolded or complained about by teachers. | □ | □ |  |
|  | d. I often get into fight with classmates. | □ | □ |  |
|  | e. I steal things. | □ | □ |  |
| 3.3. Sibling Relationships | a. I get along well with my siblings. | □ | □ |  |
|  | b. I often have serious conflicts with my siblings. | □ | □ |  |

4. **Parenting Capacity**

|  | Yes | No | Remarks |
| --- | --- | --- | --- |
| a. My caregiver often forgets where I am or what I'm doing. | □ | □ |  |
| b. I am often at home alone, without any adults. | □ | □ |  |
| c. I have had several different caregivers recently. | □ | □ |  |

5. **Abuse/Neglect**

|  |  | Yes | No | Remarks |
| --- | --- | --- | --- | --- |
| 5.1. Physical Abuse | a. In the past two months, I have been hit by a caregiver or family member because I did something wrong, did something unsatisfactory, or for no reason at all. | | | |
|  | □ Yes, the person has:  □ Spanked my buttocks with a hand  □ Hit my buttocks with an object  □ Hit my hands, arms, legs, or other body parts with a hand  □ Hitting the child's hands, arms, legs, or other body parts with an object  □ Pinched me  □ Shaken me  □ Other: ＿＿＿＿＿＿＿ | | □ |  |
| 5.2. Psychological Abuse | a. People at home often say insulting or hurtful things to me (e.g., "stupid," "lazy," "ugly"). | □ | □ |  |
|  | b. I have always felt that someone at home dislikes me. | □ | □ |  |
|  | c. Someone has said to me, "No one wants you." | □ | □ |  |
|  | d. I am often compared negatively to the incarcerated parent/not living at home. | □ | □ |  |
| 5.3. Sexual Assault | a. I feel/suspect that I have been sexually abused or forced to do things related to sex. | | | |
|  | □ Yes, including the following:  □ Someone touched me sexually or made me touch them sexually.  □ Someone has wanted me to do or watch things related to sex.  □ Someone has sexually assaulted me.  □ Someone threatened to harm or slander me unless I do sexual things with them.  □ Someone forced me to have sex with them.  □ Someone told me dirty jokes that I did not want to hear.  □ I have been forced to watch pornographic videos or images.  □ Other: ＿＿＿＿＿＿＿ | | □ |  |
| 5.4. Neglect (Emotional) | a. I have never felt loved. | □ | □ |  |
|  | b. I feel I don’t really count as part of the family. | □ | □ |  |
| 5.5. Neglect (Physical) | a. My caregiver provides me well with basic needs like food, clothing, and medical care. | □ | □ |  |
|  | b. When I need it, I'm not sure if anyone will come to protect me. | □ | □ |  |

6. **Matters Related to Early Juvenile Delinquency**

|  | Yes | No | Remarks |
| --- | --- | --- | --- |
| a. I lose my temper when I am frustrated. | □ | □ |  |
| b. I break things when I am frustrated. | □ | □ |  |
| c. I hit people when I am frustrated. | □ Yes, because  ___________________ | □ |  |

7. **Perceived Stigma**

|  | Yes | No | Remarks |
| --- | --- | --- | --- |
| a. I have felt looked down upon or mocked by certain authority figures/teachers. | □ | □ |  |
| b. I have been rejected by classmates, friends, neighbors, relatives, or professionals. | □ Yes, because  ___________________ | □ |  |
